# Anaerobic Biodegradability of Commercial Bioplastic Products: Systematic Bibliographic Analysis and Critical Assessment of the Latest Advances

**DOI:** 10.3390/ma16062216

**Published:** 2023-03-09

**Authors:** Marica Falzarano, Alessandra Polettini, Raffaella Pomi, Andreina Rossi, Tatiana Zonfa

**Affiliations:** Department of Civil and Environmental Engineering, University of Rome “La Sapienza”, 00184 Rome, Italy

**Keywords:** anaerobic digestion, biopolymers, PHA, PHB, PLA, starch-based, Mater-Bi, cellulose-based, PBAT, PCL

## Abstract

Bioplastics have entered everyday life as a potential sustainable substitute for commodity plastics. However, still further progress should be made to clarify their degradation behavior under controlled and uncontrolled conditions. The wide array of biopolymers and commercial blends available make predicting the biodegradation degree and kinetics quite a complex issue that requires specific knowledge of the multiple factors affecting the degradation process. This paper summarizes the main scientific literature on anaerobic digestion of biodegradable plastics through a general bibliographic analysis and a more detailed discussion of specific results from relevant experimental studies. The critical analysis of literature data initially included 275 scientific references, which were then screened for duplication/pertinence/relevance. The screened references were analyzed to derive some general features of the research profile, trends, and evolution in the field of anaerobic biodegradation of bioplastics. The second stage of the analysis involved extracting detailed results about bioplastic degradability under anaerobic conditions by screening analytical and performance data on biodegradation performance for different types of bioplastic products and different anaerobic biodegradation conditions, with a particular emphasis on the most recent data. A critical overview of existing biopolymers is presented, along with their properties and degradation mechanisms and the operating parameters influencing/enhancing the degradation process under anaerobic conditions.

## 1. Introduction

In the last decades, plastic pollution has become a global issue and a threat to the environment and human health. World plastic waste production is close to 400 Mt/y and the recycled share is 9% [1]. The remaining part of plastic waste is incinerated (19%) or landfilled (50%), diverting potentially valuable materials from recycling or recovery. Relatively low materials and energy recovery rates are mainly related to technical and economic constraints that limit the feasibility of the valorization processes.

Another critical aspect of plastic waste management is represented by its uncontrolled dispersion into the environment, which accounted for 22 Mt in 2019 [1]. Oceans are the ultimate sink for plastic debris, with an estimated annual input of 4.8–12.7 Mt [2]. Due to their recalcitrant nature, fossil-based plastics accumulate in the environment, and in particular in oceans, where they group into giant floating plastic islands. The main issues related to dispersion of plastic waste involve, on one hand, the potential release of hazardous chemical substances, and on the other hand, their physical disintegration into smaller particles [3], which may even be more dangerous. Microplastics can accumulate persistent organic contaminants and metals due to their high surface area and can enter the food chain, representing a hazard to living organisms [4,5].

In an attempt to enhance the circularity of the plastic sector, the main steps to take include the reduction, reuse, and recycling of plastics, as dictated by the European Circular Economy Action Plan [6]. Another emerging strategy involves replacing commodity plastics with bioplastics. This new category of materials has already been successfully employed to replace plastics in many industrial applications, and especially in the packaging sector [7].

The main advantage of biodegradable plastics is that they can be treated together with the organic fraction of municipal solid waste using the already existing infrastructure for collection and treatment. In particular, anaerobic treatment could help meet the growing demand for energy, while lowering the carbon footprint of waste management [8,9]. Bioplastic residues could positively affect the energy recovery of anaerobic digestion plants, as was reported by Cucina and colleagues [10], who co-digested sewage sludge and bioplastics and found a 45% increase in methane production compared to sludge mono-digestion. A synergistic effect in bioplastics and biowaste co-digestion was observed by other authors as well [11,12].

However, there are many issues related to the actual biodegradation profile of bioplastics which have not yet been comprehensively addressed by the scientific community [13,14]. For example, the correlation between the chemical composition of the products and their actual biodegradation is still unclear, as are the potential generation of undesired degradation products (including micro-bioplastics) and their effect on the final compost and digestate quality. This issue is of particular relevance with regard to sanitary issues, since contaminated compost and digestate may become carriers of recalcitrant substances across the environmental compartments [15]. Understanding the material-related and environment-related aspects that determine the actual biodegradation of bioplastics is necessary to harmonize their treatment with biowaste using the typical processing conditions of waste treatment plants [10].

Another issue is the regulation of the bioplastic industry, which still needs to be drafted and implemented. Currently, there are no harmonized indications on bioplastics composition, minimum content of bio-based components, nor labelling standards. The European Union is currently heading towards defining some ground rules and has recently stated that bioplastics products should only be used provided they are useful to increase biowaste capture and avoid contamination [16]. On the other hand, litter-prone items, which have been also identified by the Directive on single-use plastics [17] are not intended to be environmentally sustainable *per se*, but it is still unclear whether they should be banned even when biodegradable.

Evidently, some intersectional work is needed involving the scientific community (to assess the characteristics and behavior of bioplastics under controlled and uncontrolled conditions), Governments and supranational organizations (to provide guidelines, policies and regulations), and the industrial and economic sectors (for the implementation of the required measures) in order to build a sustainable and circular value chain of bioplastic materials.

## 2. Bioplastics: Definitions and Classification

Bioplastics currently represent 1% of the global plastic production capacity, with a volume of over 2 Mt per year [18].

Three main categories of bioplastics can be identified based on their composition and biodegradability [19]. The first and more controversial category includes the so-called drop-in plastics, which are biologically derived but are not biodegradable and are designed to mimic petroleum-based plastics. The precursors used in the production of this kind of plastic rely on agriculture; hence, they are competing with food production [20]. Moreover, the lack of degradability poses a limitation to the residues management, hindering materials recovery. Some examples of these plastics are bio-ethylene, bio-polyethylene (bio-PE), bio-propylene (bio-PP), and bio-polyethylene terephthalate (bio-PET). Some fossil-based plastics, such as polycaprolactone (PCL), polybutylene succinate (PBS), and polybutylene adipate terephthalate (PBAT), are recognized to be biologically degradable and are extensively used in the bioplastics industry. However, their production relies on fossil fuels and they usually display lower degradation rates due to their unfavorable physical and chemical characteristics [21].

Bio-based and biodegradable plastics are derived from renewable sources, such as biomasses (polylactic acid [PLA], starch) or microorganisms’ intracellular reservoirs (polyhydroxyalkanoates (PHAs)) and can be fully mineralized into harmless compounds.

Each of these biopolymers has a specific chemical structure, degree of crystallinity, and associated physical, mechanical, and thermal properties that, in turn, determine the type of use they are more suited to. Biopolymers can be classified according to different criteria, including, e.g., polymer nature, thermal behavior, origin, and biodegradability characteristics. Some common categories include:

- Bio-based aliphatic polyesters (PLA, PBS, PHAs);
- Cellulose-based bioplastics;
- Starch-based bioplastics;
- Bio-based aromatic polyesters (polyethylene furanoate, PEF);
- Bio-based polyurethanes;
- Fossil-derived biodegradable polymers (PVA, PBAT, PCL, Polyglycolic acid, PLGA).

Another classification may be made on the basis of the origin of the polymer [22], distinguishing among artificially processed and microbially and naturally derived materials. Examples of artificially processed-type plastics include PLA and PBS. Microbially derived bioplastics comprise different types of PHAs. Examples of naturally derived bioplastics may include starch coalesced either with esters or cellulose.

In the following sections, a description of the relevant characteristics of the main bioplastic materials is provided.

### 2.1. PHAs

PHAs are a class of biopolyesters synthesized and accumulated intracellularly by numerous microorganisms [23,24], particularly under cell stress conditions (typically, presence of excess carbon and limitation of essential nutrients [25]). During such conditions, microorganisms divert their metabolism, instead of cell duplication, towards the formation of hydroxyalkyl-CoA, a precursor of PHA polyesters [26], which, in turn, are stored as internal cellular reserves of carbon and energy. Under starvation conditions, these reserves are then used to sustain the main metabolic functions of microbial cells. PHAs have the capability of being stored at high concentrations (up to 90% of cell dry weight for specific pure cultures [26]) within the cell cytoplasm since they are known to produce no significant changes in osmotic pressure. After the accumulation stage, microbial cells can be harvested and PHAs extracted through different techniques.

PHAs have attracted considerable scientific interest owing to their thermoplastic and elastomeric properties, as well as to their biodegradability and biocompatibility. Furthermore, they can be synthesized biochemically from a wide variety of residual organic feedstocks, particularly those that are suited to fermentation, yielding volatile fatty acids which are the common starting substrate for PHA production.

The different known chemical structures of PHAs differ by the number of carbon atoms of the constituting monomer, and can be classified as short-chain (3–5 carbon atoms) or medium-chain (6–14 carbon atoms) PAHs [27]. The most common polymers belonging to this family are poly(3-hydroxybutyrate) and poly(4-hydroxybutyrate) (PHB), poly(3-hydroxybutyrate-co-3-hydroxyvalerate) (PHBV), poly(3-hydroxybutyrate-co-4-hydroxybutyrate), and poly(3-hydroxybutyrate-co-3-hydroxyhexanoate). PHB is the most studied and commercialized, mainly for packaging and biomedical applications [28].

### 2.2. TPS

Starch is a polysaccharide derived from plants and mainly composed by amylose and amylopectin, which can be found in different proportions and determine the polymer properties [29]. Starch is particularly widespread thanks to its availability and low cost [30], but has poor tensile properties and a high hydrophilic nature, so it is turned into thermoplastic starch (TPS) to achieve a better processability [31]. The disruption of starch granules is performed through gelatinization and the addition of water and glycerol as a plasticizer [32]. In addition, to obtain the required physical and mechanical properties, TPS is often blended with other polymers or additives [33]. Many different inclusions are used to reinforce the material and improve its gas barrier capacity, such as fibers [34,35] and clay fillers [36] or metal oxides [37].

### 2.3. PLA

PLA is an aliphatic polyester obtained from renewable resources. It is produced through direct polycondensation of lactic acid or via ring opening polymerization of lactide [38] and can differ depending on the relative proportions of the two stereoisomers of lactic acid, which are D- and L-lactide [39,40]. PLA is one of the most successful biodegradable polymers since it is already employed for many different industrial applications, particularly for packaging and in biomedicine [7]. Given its brittle behaviour, it is often co-polymerized and blended with additives to improve its mechanical and physical properties [41,42,43,44].

### 2.4. PCL

Poly (ε-caprolactone) is an alyphatic polyester usually obtained from the ring opening polymerization of ε-caprolactone [45]. It belongs to the category of fossil-based and biodegradable plastics and, thanks to its biocompatibility and slow degradability, it is frequently used for biomedical and packaging applications [46,47]. It is a semi-crystalline and hydrophobic polymer, with a melting point in the range 59–64 °C. When blended to other polymers (mainly starch and PLA) it shows a good compatibility and is used especially due to its thermoplastic behavior, which helps the processing of the material [48].

### 2.5. PBS

PBS is an aliphatic and thermoplastic polyester, which is derived through polycondensation of succinic acid or dimethyl succinate and 1,4 butanediol [49]. The production process may include either ring-opening polymerization or enzymatic polymerization; the starting monomers are commonly petroleum-based but recent advances have also been made towards PBS production from bio-based sources [50]. PBS displays good processability, good tensile and impact strength, as well as a lower production cost compared to PLA and PCL [51]. However, its mechanical and physical characteristics do not often meet the requirements for a number of industrial applications, since it is distinguished by moderate rigidity and poor gas barrier properties [52] due to its low glass transition temperature that makes it unsuited for use for rigid packaging production [50]. Additives and fillers, as well as blending with other polymers, have been studied to enhance its mechanical and physical properties [53,54].

### 2.6. PBAT

PBAT is an aliphatic-aromatic polyester produced by poly-condensation of butanediol, adipic acid, and terephthalic acid [55]. Its degradability is mainly governed by the aliphatic part of the polymer [56], while the aromatic chain determines the typically good mechanical properties of the material that make it suitable for many applications, such as high ductility and processability [57]. PBAT has been widely studied in blends, especially with PLA [57,58,59].

## 3. Bioplastics Biodegradation

### 3.1. General Concepts and Influencing Factors

Biodegradation of organic matter involves microbially mediated conversion of the original compounds into water, biomass cells, CO_2_ (under aerobic conditions) or CO_2_, CH_4_, and minor amounts of other gaseous products (under anaerobic conditions).

The process can occur in natural environments under uncontrolled conditions or in dedicated systems where the operating parameters, the process factors, and the metabolic products can be monitored more easily.

Based on the current state of the art, most biodegradable plastics are engineered to be degraded in aerobic environments, which has fostered a large quantity of scientific studies on the assessment of the aerobic degradability of such materials. On the other hand, the research about the biodegradation features of commercial bioplastic products under anaerobic conditions has only very recently developed systematically. As a result, definitive conclusions on the degree of anaerobic biodegradability, the governing mechanisms, and the influence of key factors are still far from having been achieved.

The anaerobic degradation of organic matter has been intensively explored over the past three decades to elucidate the underlying biochemical pathways, the microbial species involved, the reaction products, as well as the main influencing factors of the process. Anaerobic digestion is a complex biochemical process resulting from the synthrophic activity of an array of microbial species having different functions and physiology, metabolic capabilities, and operating conditions requirements. Such microorganisms, therefore, play a specific role in one of the sequential process phases (hydrolysis, acidogenesis, acetogenesis, and methanogenesis). In general, and particularly for complex substrates such as the polymeric structures of bioplastics, hydrolysis—which involves the breakdown of the original substrate molecules into simpler species that can be further metabolized by the microorganisms—is recognized to be the rate-limiting step of the whole process and is therefore crucial for the subsequent biochemical pathways. Acidogenic microorganisms convert the hydrolyzed compounds into short-chain fatty acids, lactate, alcohols, and chetons. These are in turn further transformed by acetogenic microorganisms into H_2_, CO_2_, and acetate; this can also be synthesized by autotrophic homoacetogens directly from the H_2_ and CO_2_ generated in the previous stage. The final methanogenic stage mainly involves the formation of CH_4_ and CO_2_ through either the acetoclastic or hydrogenotrophic pathways [60,61]. The main microbial species taking part in the process include hydrolytic bacteria, primary/secondary fermentative bacteria, and methanogenic archaea, which are synthrophically connected through the exchange of H_2_, formate (as electron carriers), and other metabolites such as acetate [62] to sustain the related microbial reactions.

Anaerobic digestion is commonly regarded as a valuable and sustainable strategy to recover materials (compost, digestate, nutrients) and energy from wastes [63,64], while at the same time contributing to reducing the net emissions of greenhouse gases from waste treatment. With regard to such aspects, anaerobic digestion can represent a valuable technological option for the management of end-of-life bioplastics, assuming that they are collected and managed together with the organic fraction of municipal solid waste. Optimized anaerobic degradation conditions—as for other biological processes—require well-balanced amounts of carbon and nutrients. Since it is well recognized that typical substrates for anaerobic digesters, such as food/kitchen waste, the organic fraction of municipal solid waste, and sewage sludge, have a typically low C/N ratio while most bioplastics are poor in nitrogen, the co-digestion of such materials may be an operating strategy to adjust the C/N ratio to optimize the digestion condition and enhance the degree of substrate conversion into biogas [11].

The estimation of biodegradability is commonly made on the basis of the volume of biogas evolved. Under aerobic conditions, the CO_2_ volume is used as an index of assimilation and mineralization of the substrate and biodegradability is expressed as the ratio between the evolved CO_2_ and the theoretical amount of CO_2_ expected (Equation (1)). Under anaerobic conditions, biodegradability is usually quantified from the ratio between the total biogas (CH_4_ + CO_2_) produced and the corresponding theoretical amount of biogas expected (Equation (2)), or as the equivalent ratio for methane instead of total biogas (Equation (3)). Equation (3) is sometimes preferred over Equation (2) since CO_2_ is relatively water-soluble (especially under elevated CO_2_ partial pressures as in digesters’ headspace); therefore, the quantification of the total biogas volume evolved requires direct determination of the dissolved inorganic carbon that should be made without altering the thermodynamic and chemical conditions of the system.

(1) $$Biodegradation\;\left(\%\right)=\frac{{\mathrm{CO}}_{2}}{{\mathrm{ThCO}}_{2}}\times 100$$

(2) $$Biodegradation\;\left(\%\right)=\frac{{\mathrm{CH}}_{4}+{\mathrm{CO}}_{2}}{\mathrm{Th}\left({\mathrm{CH}}_{4}+{\mathrm{CO}}_{2}\right)}\times 100$$

(3) $$Biodegradation\;\left(\%\right)=\frac{{\mathrm{CH}}_{4}}{{\mathrm{ThCH}}_{4}}\times 100$$

The theoretical volumes of CO_2_ and biogas produced are calculated from the polymer’s carbon content under the hypothesis that this is totally converted into the final products, e.g., neglecting the amount of carbon incorporated in the microbial cells due to biomass growth. For instance, under anaerobic conditions, the Buswell equation is commonly adopted (Equation (4)) [65]:

(4) $$C_{n}H_{a}O_{b}+\left(n-\frac{a}{4}-\frac{b}{2}\right)H_{2}O\to \;\left(\frac{n}{2}+\frac{a}{8}-\frac{b}{4}\right){\mathrm{CH}}_{4}+\left(\frac{n}{2}-\frac{a}{8}+\frac{b}{4}\right){\mathrm{CO}}_{2}$$

It should be considered that the Buswell equation does not take into account the substrate conversion into biomass; therefore, the actual biogas production has an upper limit that is obviously lower than that expected from Equation (4) [66].

Biodegradation is a process governed by the combination of different factors, depending on the polymer characteristics and on the environmental conditions it is subjected to.

The configuration of the monomeric units constituting the polymer, the bonds among the elements, and their orientation dictate the material properties, which, in turn, influence its biodegradation profile. In general, the presence of hydrolyzable groups in biopolymers (ether, ester, amide, and carbonate) is the factor that determines their susceptibility to microbial attack [67]. The solubility of polymers typically decreases as the polymeric chain length and molecular weight increase. Crystallinity improves water resistance, therefore limiting both hydrolysis and the microbial activity that are instead favored in amorphous regions. On the other hand, hydrophilicity determines higher vulnerability to water.

Flexibility is another characteristic that lowers the degradation enthalpy since it improves the possibility to fit better into the active sites of enzymes. Aliphatic polyesters have, in general, a larger flexibility compared to the aromatic and aliphatic-aromatic counterparts and are therefore particularly suited for degradation [68].

Polymers with lower molecular weights, a higher amorphous character, and higher flexibility are in principle more prone to biological attack [69].

Furthermore, exposure conditions to potential degradation agents/factors can complement polymers characteristics and improve degradability. The main external factors affecting biodegradation can be both biotic and abiotic. Each environment typically has a specific microbial community and the main abiotic factors, such as temperature, pH, and moisture, can promote their growth and activity [70].

Biodegradation is an enzymatic reaction and proceeds very specifically depending on the chemical bonds/linkages of the polymer and the structure of particular functional groups. In general, microorganisms are only capable of attacking specific functional groups at specific sites.

Temperature has an effect on enhancing the hydrolysis and the overall process rate [71] by increasing polymer chains mobility and enzymatic activity. When temperature is in the range of the polymer’s T_g_, the material becomes more flexible. Acidic or basic environments have been found to accelerate hydrolysis as well. Of course, moisture is involved in the hydrolysis of polymeric materials as well as in sustaining microbial activity. Another mechanism of biopolymer alteration involves photodegradation, which depends on the interaction between the polymer and UV radiation.

### 3.2. Biodegradation Mechanisms

**Figure 1:**
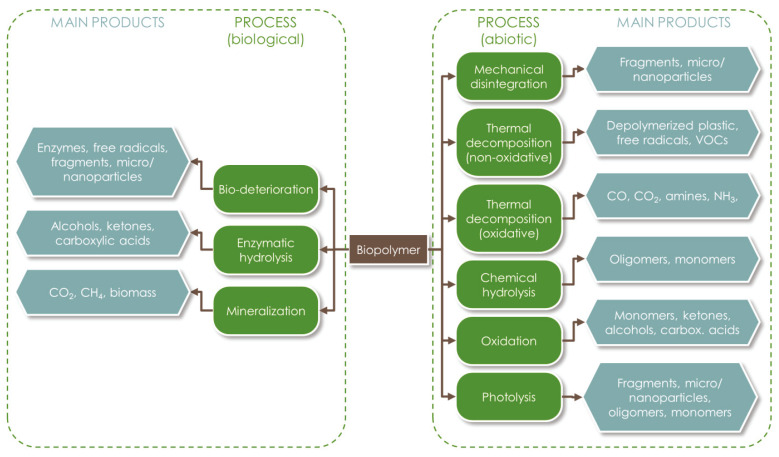
Main abiotic and biotic degradation mechanisms of biopolymers and related products.

Polymers biodegradation is the result of the competition and combination of multiple mechanisms. As illustrated in Figure 1, both abiotic and biotic (enzymatic) actions can lead to the cleavage of the polymer’s chemical bonds, and later to matrix erosion [47]. The process can be carried out at different levels: surface level, bulk level, or through autocatalysis [45]. Surface degradation is a heterogeneous process which may also be detected visually, while bulk erosion affects the whole matrix at the same time, so that the material remains apparently the same for a long time until it disaggregates abruptly [72]. Bulk erosion is more related to the influence of abiotic factors, which may include mechanical stresses (resulting from compression, tension, or shear forces), thermal alteration, water absorption, chemical hydrolysis, oxidation, or photolysis [73,74]. The resulting fractures can favor the microbial degradation pathways. Autocatalysis is a phenomenon that happens internally, where the oligomers and monomers released remain trapped into the matrix and are able to continue cleaving the polymeric backbone from the inside. Regardless of the mechanisms involved, the degradation of the polymeric matrix can be tracked with the monitoring of molecular weight and monomers release [72].

In general terms, the main steps in the degradation of polymers include: (i) biodeterioration; (ii) depolymerization; (iii) assimilation; and (iv) mineralization [75]. Biodeterioration causes changes in the physical, mechanical, and chemical characteristics of the material. It begins with the adhesion of microorganisms on the material surface and the formation of a biofilm. Extracellular depolymerase enzymes and free radicals are generated and their action leads to the formation of cavities, microfractures, and the cleavage of the polymer backbone. A physical surface embrittlement and bulk erosion may also complement the enzymatic degradation, increasing the material’s surface area exposed to microbial attack, thus promoting the subsequent biodegradation reactions. In this phase, hydrolysis occurs thanks to the diffusion of water into the amorphous regions of the polymeric matrix. For instance, the butylene adipate and butylene terephthalate components of PBAT degrade at different rates, with the former being less crystalline [56]. Moreover, the kinetics of this process depend on the polymer hydrophilicity; thus, it is generally very slow for PCL [76].

Depolymerization and assimilation are carried out by two categories of enzymes that are extracellular and intracellular. Extracellular enzymes are secreted by microorganisms and can act randomly on the disruption of specific bonds or linkages in the polymeric structure, releasing intermediate metabolic products with simpler molecular structures, with an associated reduction in the molecular weight of the material [71]. Some authors observed that the efficacy of enzymatic hydrolysis is dependent on the degree of adsorption of the enzyme onto the polymer surface, which is the pre-condition required for surface erosion of the polymer [77].

Extracellular enzymes exert their action according to two different polymer cleaving modes: endo-type hydrolysis involves random scission of ester bonds along the main chain of the polymer, releasing either monomers or short-chain soluble oligomers; on the other hand, in exo-type hydrolysis, the material is degraded stepwise from the chain ends of the polymeric structure (for instance, either the hydroxyl or the carbonyl end of the molecule in the case of polyesters), with oligomers being mainly generated at first by the cleavage action [78].

In particular, the ester bond in the polyesters’ backbone is susceptible to non-enzymatic scission that occurs through the following reaction [79]:

$$-\mathrm{COO}-+H_{2}O\to -\mathrm{COOH}+{\mathrm{OH}}^{-}$$

The formation of carboxylic groups, in particular, determines the further autocatalysis of the breakage of ester linkages, since polymer oligomers have a lower pKa compared to most carboxylic groups [79,80]. In PBAT, the cleavage of ester linkages is coupled with the reaction between water and the carbonyl groups located in the proximity of the benzene rings [56].

The type of intermediate metabolites produced in the depolymerization phase depends on both the specific polymer of concern and the type of enzymes involved [81].

It was observed that PLA degradation into lactic acid oligomers begins when a molecular weight drop to below 10,000 Da [79] and the main enzymes involved are proteases and lipases [82,83]. The same enzymes were found to be responsible for PCL ester bond cleavage [47]; as a result of such bond breaking, the polymer is broken down to carboxyl terminal groups and 6-hydroxylcaproic acid [45].

During degradation of PBS, degrading enzymes including esterases, lipases, and cutinases were identified [50,78,84]. Exo-type cleavage was observed in the presence of lipase, with 4-hydroxybutyl succinate dimer as the main hydrolysis product by some investigators [77,78]. In another study [85], an enzyme extracted from *Aspergillus* sp. was found to be capable of degrading PBS, again through exo-type hydrolysis at the carboxylic chain end; in this case, the degradation products were found to include succinic acid, butylene succinate, succinic acid-butylene succinate, and their salts. PBS degradation using cutinase was tested in a number of studies [84,86] that revealed endo-type hydrolysis of the polymer, although different chain scission modes (either at the hydroxyl or at the carbonyl end of the polymer) were found to occur based on the observed degradation products.

A series of enzymes (hydrolase, lipase, esterase, and cutinase) were identified in both composting and anaerobic digestion environments in PBAT degradation [87], with the subsequent production of terephthalic acid, adipic acid, and 1,4-butanediol [88].

PHB and PHBV were found to be broken down by depolymerases and hydrolases to 3-hydroxybutyric acid and both 3-hydroxybutyric acid and 3-hydroxyvaleric acid, respectively [27].

During starch degradation, the amylose and amylopectin acetal links are hydrolyzed by amylase and glucosidase, respectively, which generate glucose, maltose, and maltotriose [89,90].

After depolymerization, long- and short-chain oligomers and soluble monomers released are able to cross the cell membranes and can then be directly exposed to the assimilation reactions, which are catalyzed by intracellular enzymes [91]. They are used by the microorganisms in both catabolic and anabolic reactions to generate energy and other metabolic products and synthesize new microbial cells. The last stage of the biodegradation process, i.e., mineralization, involves the final substrate conversion into water, biomass cells, CO_2_ (under aerobic conditions) or CO_2_, CH_4_, and minor amounts of other gaseous products (under anaerobic conditions).

### 3.3. Microbiology of Bioplastics Biodegradation

The specific type of microbial pathways occurring and the related microbial species involved are crucial for the degradation of the polymeric matrix of bioplastic products. More than 90 types of microbes were found to be involved in bioplastics degradation [69], mainly deriving from compost or soil environments. Currently, little is known on the specific role of each microbial species in the biodegradation process, particularly regarding anaerobic conditions [92,93]. In general terms, the microorganisms found in anaerobic digesters are mainly bacteria; archaea are present as well and take part in the methanogenic phase [94].

The operating temperature has a large influence on the microbial community development. During mesophilic treatment of bioplastics, a prevalence of *Bacteroidota*, *Chloroflexi*, *Desulfobacterota*, *Firmicutes*, and *Euryarchaeota* was observed, while at thermophilic temperatures, *Firmicutes*, *Proteobacteria*, and *Coprothermobacter* were found to be predominant [94,95]. Increased temperatures were also observed to favor the growth of hydrogenotrophic methanogens [96]. Some attempts have been made at isolating bacterial strains, which were also found to become more efficient as the degradation time was reduced [97,98].

A number of authors attempted to identify the microbial strains participating in the degradation of specific bioplastic matrices. For starch-based products, a prevalence of *Firmicutes* and *Synergistetes* operational taxonomic units (OTUs) was observed under thermophilic conditions, while a dominance of *Bacteroidetes*, *Firmicutes*, *Chloroflexi*, and *Proteobacteria* was detected under mesophilic conditions [96].

PHB was found to be degraded by the genus *Clostridium botulinum* [97] and by consortia of *Ilyobacter delafieldii*, *Enterobacterm* and *Cupriavidus* [99]. Moreover, Yagi and colleagues tested PHB and detected *Arcobacter thereius* and *Clostridium* sp. when operating under mesophilic temperatures [100], and Peptococcaceae bacterium Ri50, *Bacteroides plebeius*, and *Catenibacterium mitsuokai* at thermophilic temperatures [101].

Several studies on PLA anaerobic degradation also reported the main microbial strains detected during the process. In many cases, lactic acid bacteria were observed, such as *Moorella*, *Tepidimicrobium*, *Thermogutta* [95,99,102]. When treating the polymer under mesophilic conditions, *Xanthomonadaceae bacterium* and *Mesorhizobium* sp. were detected [100], while *Ureibacillus* sp. was identified under thermophilic conditions [101]. *Methanosaeta*, *Methanoculleus*, and *Methanobacterium* were the methanogenic archaea mainly found during the anaerobic degradation of PLA [100,103].

PCL was found to be degraded by strains of the *Clostridium* genus [97] and *A thereius* [100], although there were also other reported cases in which PCL displayed a remarkable resistance to microbial attack under anaerobic conditions compared to compost or soil environments [68,97].

The understanding and control of the microbial consortia operating during the anaerobic degradation process may be used to maximize substrate conversion and the related biogas production. Molecular biology techniques could be used as a tool to this aim. In the past years, many attempts have been made to improve bioplastic production processes through the use of modified enzymes by protein engineering [104], while investigation on applications to enhance bioplastic degradation is still in its infancy. However, enzymatic degradation of bioplastics could represent a viable option if correctly assessed and standardized [105]. Bioaugmentation may also be a useful tool; however, so far, it has been explored mainly for composting conditions. For instance, Mistry and colleagues tested high molecular weight PLA films with an ad hoc degrading bacterial consortium with *Nocardioides zeae* EA12, *Stenotrophomonas pavanii* EA33, *Gordonia desulfuricans* EA63, and *Chitinophaga jiangningensis* EA02 and observed a 50% increase in mineralization compared to the test with indigenous microorganisms [106]. Expanding the research in the way of engineered enzymes or introducing the assessment of bioaugmentation strategies could improve the current understanding of the anaerobic degradation of bioplastics.

### 3.4. Biodegradation Monitoring Techniques

Since the degradation of biopolymers and biopolymer-based materials is a complex process, it can be monitored and assessed using different approaches and viewpoints. The assessment of biogas and methane production can be complemented with further analyses, which can provide additional information on the physical, mechanical, chemical, and microstructural characteristics of the material at different stages of degradation. The data retrieved using different approaches can then be used to derive correlations and draw more detailed conclusions on the biodegradation process.

The additional methodologies that can be used belong to five main categories, including disintegration measures, morphologic/visual inspection, microbiological characterization, thermal behavior, and spectroscopic analyses.

Disintegration can be assessed through mass loss measurements at different times to monitor the evolution of polymer disruption.

Visual inspection can be carried out at a macroscopic level by observing the plastic fragments at the end of the experiment, provided that they are still visible at the naked eye. More advanced particle observation techniques, such as optical microscopy or scanning electron microscopy (SEM), can be used to monitor the physical changes at the microscopic level.

The analysis of the microbial community involved during the degradation process can provide further information on the adaptability of microorganisms to the polymeric substrate and the compatibility of the material with the environmental conditions it was subjected to.

The analysis of the thermal behavior of the material can give an insight into the changes occurring in its physical and chemical properties. To this aim, the most used techniques are thermogravimetric analysis (TGA) and differential scanning calorimetry (DSC) that can identify key temperatures in polymer phase transitions.

Spectroscopic analysis can also be carried out using Fourier-transform infrared (FT-IR) or X-ray diffraction (XRD) techniques, which can assist the identification of major chemical bonds in the matrix and their rearrangement as a result of biodegradation.

## 4. Methods

As described and motivated in the previous sections, this paper summarizes the main scientific literature on anaerobic biodegradation of bioplastics through a general bibliographic analysis and a more detailed discussion of specific results from relevant experimental studies. The analysis of literature data on bioplastics biodegradation was deliberately restricted to anaerobic environments, since numerous very recent studies have been published on this topic.

A systematic bibliographic analysis on the subject was conducted in the Web of Science (WoS) Core Collection database, currently managed by Clarivate Analytics. This was chosen among the most commonly used and trusted databases (Dimensions, Google Scholar, Lens, PubMed, Scopus, Web of Science) for academic research in scientific and technical disciplines. The database was accessed in December 2022 and the research was refined for inclusion of the latest scientific references on 22 January 2023. The string used for data search and extraction was (biodegradation OR biodegradability OR degradability) AND (bioplastics OR bioplastic OR biopolymers OR (biodegradable AND plastics) OR PLA) AND (anaerobic OR digestion OR co-digestion OR digester OR digesters OR biogas OR biomethanization). The initial search output was then screened based on the title and abstract contents to remove non-pertinent references that may have biased the subsequent data analysis.

A first analysis of the scientific literature on the topic of concern was conducted with the main purpose of deriving some general features of the research profile, trends, and evolution in the field of anaerobic biodegradability of bioplastics. The main features addressed in the bibliographic analysis are the following:

- Volume of the scientific production in the field and its time evolution, to highlight emerging research trends on the topic;
- Geographic distribution of the scientific studies, to identify the geographic areas most concerned on bioplastics degradability-related issues;
- Research areas, to visualize the main scientific fields of investigation;
- Frequency of keywords occurrence, to pick out research hot topics;
- Co-occurrence network of keywords, to find central keywords and clusters of research themes.

The analysis of such aspects was conducted using the bibliometric mapping software tools VOSViewer version 1.6.18 [107] and Bibliometrix version 4.1 [108], as well as by custom processing of the extracted data in spreadsheet format.

A second stage of the analysis of literature data involved extracting detailed results about bioplastic degradability under anaerobic conditions. This was performed by screening suitable candidate papers for analytical and performance data on biodegradation performance for different types of bioplastic products and different anaerobic biodegradation conditions, with a particular emphasis on the most recent data (publication years: 2022 and early 2023). The information retrieved from the selected literature references was built on the data collected by three previous excellent reviews on the subject [93,109,110], expanding the dataset by including 2022 and early 2023 results along with additional data and results from further papers that had not been included in these review studies.

In some cases, data retrieval from the different reviewed publications required extracting the numerical values from the original graphical format. This was conducted using WebPlotDigitizer, a semi-automatic tool for data extraction from images of graphical data visualization [111]. In other cases, conversion of the units of measure was required to present the results as uniformly as possible. When this was not allowed due to the lack of information in the related publication, the data were kept in their original format and reported as such in the discussion.

## 5. Summary and Discussion of Literature Data on Anaerobic Degradation of Bioplastics

### 5.1. General Bibliographic Analysis

**Figure 2:**
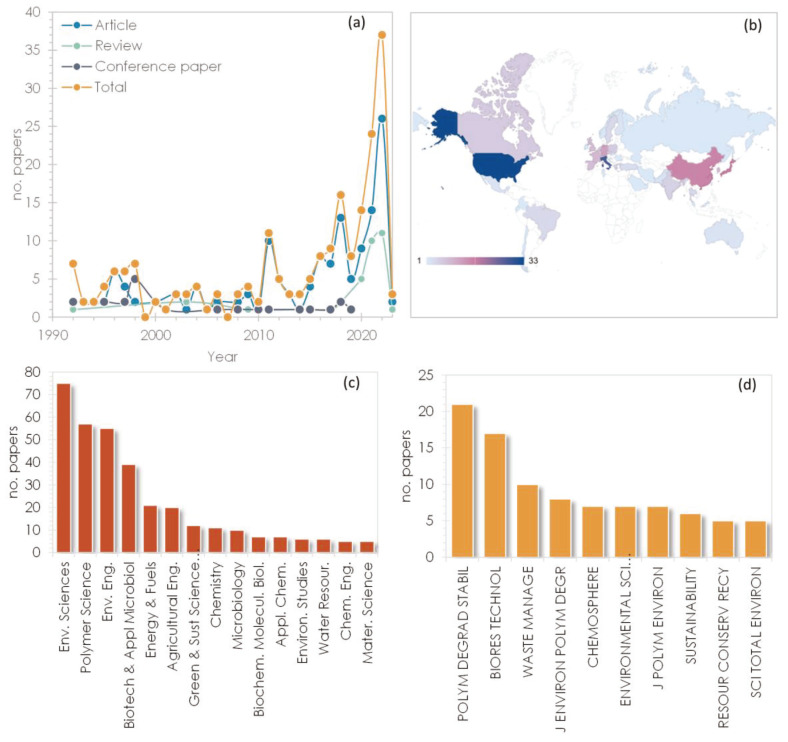
(**a**) Time evolution of published papers by article type; (**b**) main contributing countries (color shades correspond to the number of papers); (**c**) main WoS categories covered; (**d**) top productive journals (Note: only categories with ≥5 papers are included in the plots).

The initial literature search in the WoS database yielded a total of 275 scientific references, which were reduced to 206 after a duplication check and pertinence/relevance screening. The excluded literature references were mostly related to the production and effects of extracellular polymeric substances during sludge treatment as well as to studies in which the anaerobic degradation of bioplastics was merely mentioned without being dealt with in detail. The publication period for the selected references covered the time span from 1992 to early 2023 (as shown in Figure 2a), the past five years have experienced a substantial increase in the scientific interest towards the anaerobic biodegradability of bioplastics, and, in particular, the topic received considerable attention in 2021 and 2022, which also justifies the need for an updated review of the latest research findings related to the subject. The 206 articles in the dataset were published in 93 sources, including journals, conference proceedings, and books. The main contributing countries (see Figure 2b) include the USA (33 papers), Italy (28), Japan (17), China (16), and Germany (12), while additional geographic areas contributing to the scientific research on bioplastic biodegradation under anaerobic conditions covered mainly Europe, Korea, North America, and India. The main research fields covered by the literature we searched are related to the areas of environmental science and engineering, (micro)biology, biochemistry, and biotechnology, as well as polymer and materials science Figure 2c), which are also mirrored by the most productive journals in the field (Figure 2d).

**Figure 3:**
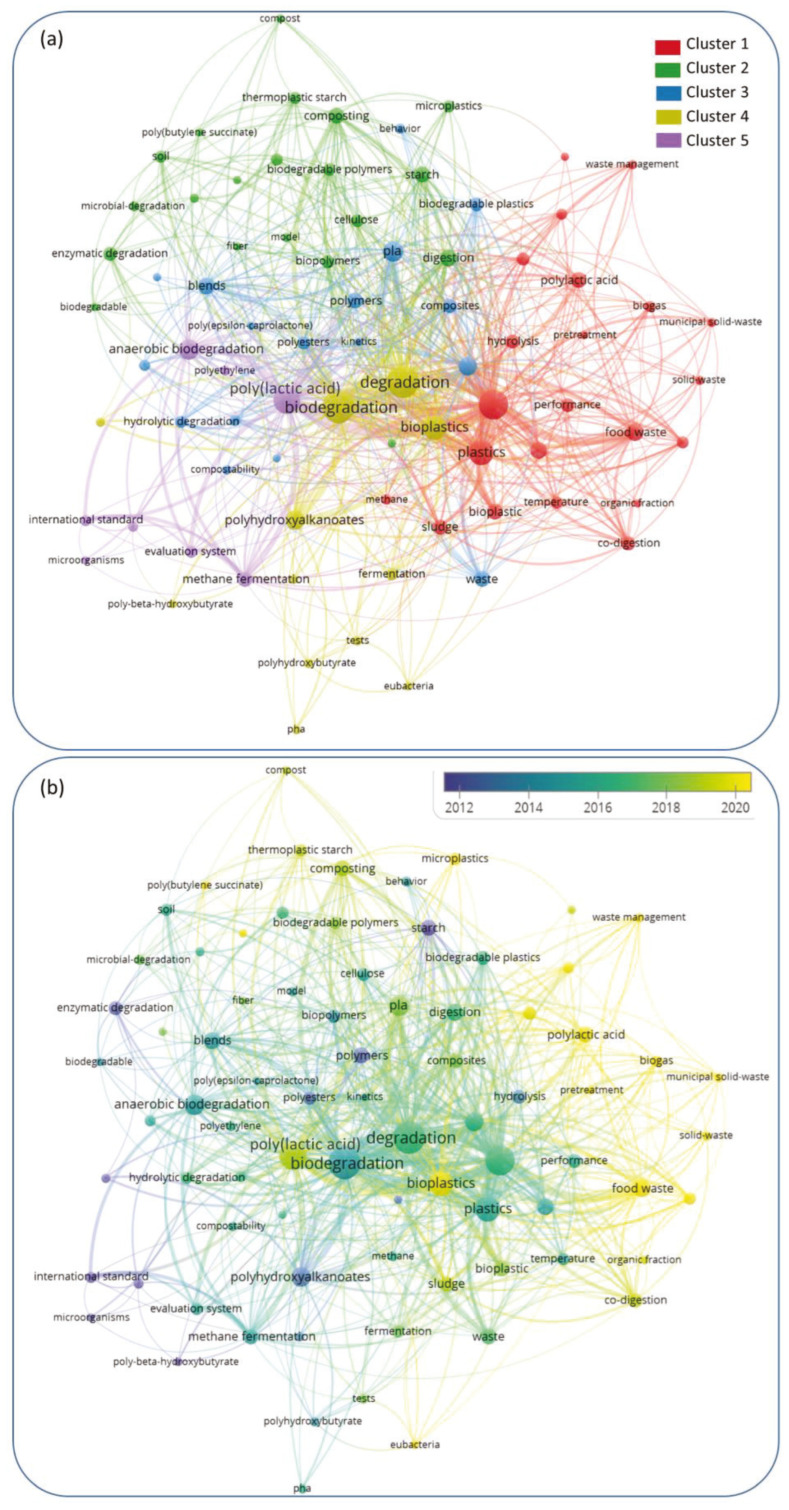
(**a**) Network of keyword co-occurrence and (**b**) overlay visualization of keyword co-occurrence over time built in VOSviewer.

The results of the analysis of keywords co-occurrence are depicted in Figure 3, where the maps report a network in which the keywords are taken as the nodes (or entities), and the links between the nodes represent the co-occurrence of pairs of keywords in the selected studies. The thickness of the links (i.e., the strength of the connection) represents the number of publications in which two keywords occur together. The network was constructed out of an overall number of 848 items, retaining only those keywords (*n* = 79) displaying a minimum number of 5 occurrences. The result of this reduction operation points out the existence of multiple issues involved in the study of bioplastic biodegradation, but also the need for standardization and homogenization of the scientific terms in the field.

The most frequent keywords were then clustered into five thematic groups (highlighted in different colours in Figure 3) based on co-occurrence so as to identify the main research areas with the investigated topic. The identified thematic clusters were explained by analyzing the subject coverage through the type and number of specific keywords used in each group. In detail, the main features of the thematic clusters resulting from the analysis can be summarized as follows:

- Cluster 1 included the main features of anaerobic digestion of bioplastics as well as co-digestion with other organic residues in the framework of waste management, with a focus on biogas production, digestion conditions, and pre-treatment;
- Cluster 2 included topics related to a comparative assessment of bioplastic degradation during composting and anaerobic digestion, modelling of the process mechanisms and kinetics as well as assessment of residual microplastics;
- Cluster 3 grouped the studies on specific bioplastic types (PCL, PLA, starch blends, composite materials);
- Cluster 4 addressed the microbial issues involved in bioplastics degradation and biopolymers generated by the fermentation of organic residues (PHA, PHB);
- Cluster 5 grouped the topics related to the evaluation of bioplastics degradability and the corresponding testing methods.

It is interesting to note from Figure 3b that the focus of the research studies on the topic has moved over the years from a more general assessment of the behaviour of specific bioplastic types and the definition of potential degradation mechanisms to the evaluation of their environmental behaviour, with particular reference to the handling and treatment of residual bioplastics in the framework of organic waste and food waste management. This is clearly due to the increasing concerns related to the effects of a massive use of bioplastic products in everyday life on the amount of waste generated and to the identification of the most suitable waste management strategies (including separate collection, treatment, and final disposal) for such materials.

### 5.2. Discussion of Literature Data

**Figure 4:**
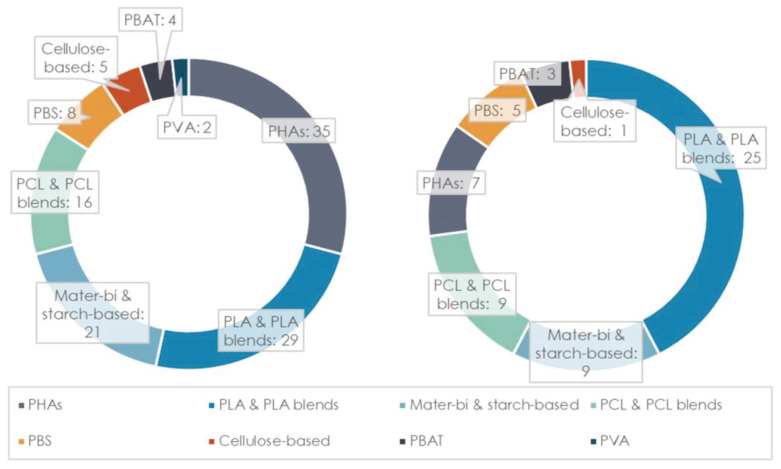
Number of studies on the different biopolymers for mesophilic (**left**) and thermophilic (**right**) conditions.

**Figure 5:**
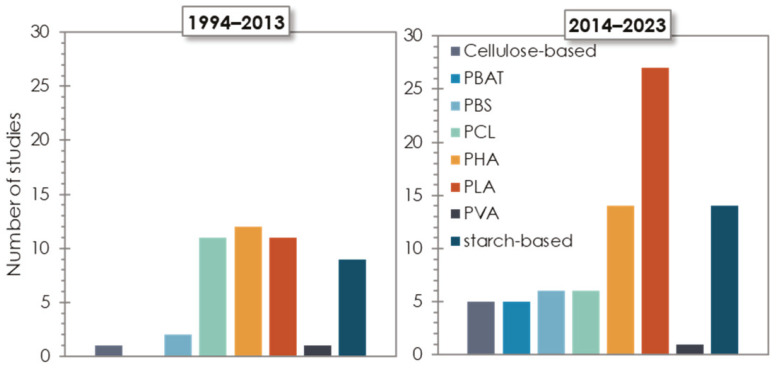
Number of studies on the different biopolymers over the last two decades.

The second stage of the analysis, based on a detailed examination of bibliographic data on the anaerobic degradability of different bioplastic products, yielded a total of 179 studies investigating biodegradation, the majority of which (120 publications) were related to mesophilic conditions, while the remaining 59 were focused on thermophilic conditions. As evident from Figure 4, the different bioplastic types have received a different level of attention by the scientific community. In particular, the biopolymers that have been most widely investigated include different types of PHAs (mainly under mesophilic conditions), PLA and PLA blends/co-polymers, and starch-based polymers (mainly Mater-Bi), followed by PCL and PCL blends/co-polymers. From inspection of Figure 5, it is also noted that the scientific interest has increased over the last decade for almost all types of biopolymers, and particularly for PLA and starch-based products, which are nowadays more widespread in commercial items.

The identified studies were reviewed to extract specific information on the testing conditions investigated (digestion temperature, amount of material tested, food-to-microorganisms ratio, biodegradation time, testing procedure), the analytical techniques used for the investigation of the biodegradation process, the observed biogas/methane production yield, and the estimated degree of biodegradation, as well as the bioplastic pre-treatment (when performed). As mentioned in the Methods section, an effort was made to report the results—whenever feasible—in a uniform way to facilitate the comparative evaluation of the information from different literature studies and allow the identification of behavioural trends or clusters among the bioplastics of concern.

The results of the detailed literature analysis are reported in Appendix A in Table A1 (mesophilic conditions) and Table A2 (thermophilic conditions). The polymers of concern were cellulose-based bioplastics, Mater-Bi and other starch-based products, TPS, various types of PHAs (PHB, PHBV, PHBO and their blends), PLA and PLA blends, PBS and PBS blends, PCL and PCL blends, and PBAT. These were investigated as either pure polymers or as commercial products (the latter presumably containing often unspecified proprietary additives and co-polymers) in different physical forms including powder, granulate, film, and whole items (plates, cups, cutlery, or coffee capsules with different mechanical characteristics).

The ranges for the digestion temperature were 30–38 °C for the mesophilic conditions and 52–58 °C for the thermophilic conditions, while the digestion time varied rather broadly across the different studies, spanning the ranges 8–520 d and 15–146 d, respectively. Bioplastic pre-treatment was also tested in a number of studies and was mainly based on thermal/hydrothermal processing, steam exposition, and alkaline or acidic hydrolysis.

The biodegradation profile of the investigated bioplastic materials was typically evaluated through Equation (3) (most commonly) or Equation (2), and in some cases was also complemented with additional data regarding the degree of material disintegration or mass loss. Further advanced characterization techniques to monitor bioplastic degradation were used in 62% of the selected literature references. Out of these, 70% used 1 or 2 additional methods, while the remaining 30% combined 3–4 different analytical techniques. In particular, among the additional characterization methods, mass loss was the most used (23% of cases), followed by morphological and visual analysis using SEM and other microscopic techniques (18%), thermal analysis (17%), and spectroscopic analysis (FT-IR, 18%). Visual macroscopic inspection of bioplastic fragments at different stages of degradation was also carried out in 12% of the studies, as was the characterization of the microbial communities involved.

**Figure 6:**
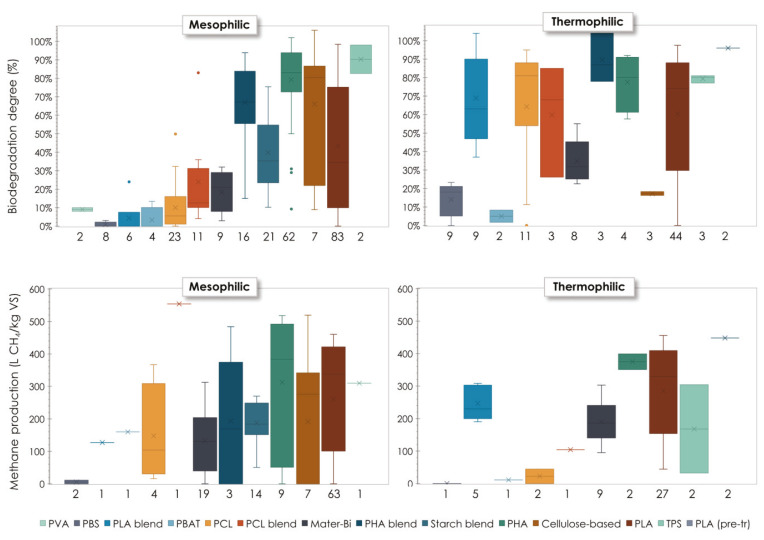
Average values (×) and range of variation (quartiles and min-max range) for the biodegradation degree and methane production yield under mesophilic (**left**) and thermophilic (**right**) conditions. Dots represent the outliers. The values below each box indicate the number of data points available.

The inspection of Table A1 and Table A2 reveal the existence of some considerable inhomogeneities throughout the specific conditions tested in the different studies in terms of digestion conditions adopted, degradation time, and approach used to monitor the degree of bioplastic conversion into biogas as well as biodegradation. As a consequence, the comparison of results from different literature sources can only be made with care, avoiding extending the conclusions beyond the validity limits of the data. Figure 6 reports the results for the estimated biodegradation degree and the observed methane production (the latter chosen based on the size of the available dataset) under mesophilic and thermophilic conditions for the different bioplastics. It should be emphasized that not all the examined studies reported both biogas/methane production and the biodegradation degree, which explains some apparent inconsistencies between the two plots that may be noted at a first glance. The box plots evidence, for all polymers, the large variability of the parameters adopted to describe biodegradability, which can be ascribed to differences in both the characteristics of the starting material (particle size, thickness, crystallinity, presence of additives, blending with co-polymers, etc.) and the specific testing conditions adopted. Notwithstanding the wide ranges of the yields of substrate conversion into methane/biogas, some general features can be identified for the investigated polymers. First, considering the mesophilic range, the materials can be grouped as follows:

- Materials displaying a generally low specific methane/biogas production and a related low degree of substrate conversion under all conditions reported in the searched literature. These include PBAT, PBS, PCL, PVA, Mater-Bi, and PLA blends, which—at least for the investigated conditions—are regarded to be poorly affected by biochemical anaerobic degradation reactions at mesophilic temperatures;
- Materials displaying typically high values of the specific methane/biogas production and the biodegradation degree. The range of polymer types belonging to this group is much narrower and includes several variants of PHAs (PHB, PHBV, PHBO, and their blends), confirming their widely demonstrated high degradability and TPS;
- Materials showing a notably variable response to anaerobic degradation, which is largely affected by the biopolymer properties and the digestion conditions as explained above. This group is made of cellulose and starch-based bioplastics as well as PLA. For these materials, the literature data are notably scattered and do not allow us to derive any conclusive general remark about their biodegradability profile.

When shifting to the thermophilic range, some polymers (PCL and PLA blends) were found to display clearly improved biodegradability, while others, such as PLA, still showed large changes in their degradation behaviour, albeit with a somewhat lower scattering of the experimental results compared to mesophilic temperatures. Most of the changes observed for such materials are related to the fact that shifting from the mesophilic to the thermophilic regime implies approaching or reaching the glass transition temperature of the polymer, at which it reduces its crystallinity and increases its hydrophilic properties, becoming, in turn, more prone to chemical hydrolysis and enzymatic degradation [75]. On the other hand, other materials such as cellulose-based bioplastics, Mater-Bi, PBAT, and PBS were found to be hardly biodegradable even at elevated temperatures.

With a view to the potential implementation of anaerobic digestion for energy recovery from bioplastic materials, the collected data show that the best methane production yields under mesophilic conditions were of the following orders of magnitude (average values for the available data sets): 260 L CH_4_/kgVS for PLA, 310 L CH_4_/kgVS for TPS, 355 L CH_4_/kgVS for cellulose-based bioplastics and 381 L CH_4_/kgVS for various types of PHAs. For the thermophilic regime, the highest conversion yields into methane were 168 L CH_4_/kgVS for TPS, 285 L CH_4_/kgVS for PLA (which raised to 448 when PLA was pre-treated to promote the hydrolysis phase) and 375 L CH_4_/kgVS for different PHA species. These results show that energy exploitation from bioplastic materials is technically feasible for selected types of polymers. The large ranges of variation of the biogas production yields reported in Figure 6 also show that there is some considerable room for improvement of the degree of substrate conversion into biogas by adequate adjustment of the polymer composition and digestion conditions. On the other hand, for the bioplastic materials for which low biogas production yields are reported, anaerobic digestion does not currently represent a viable treatment option, unless their biodegradability profile is remarkably improved through either proper design of the blend composition or the application of suitable pre-treatment processes.

**Figure 7:**
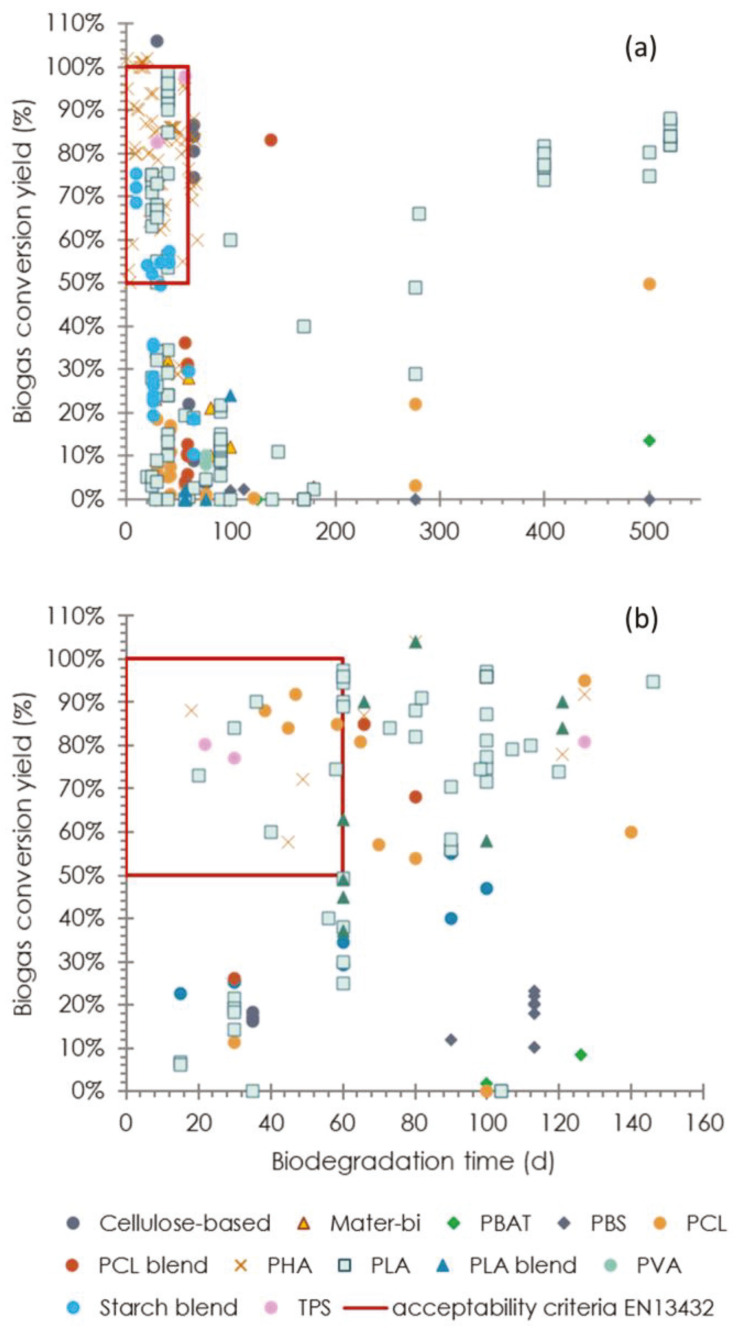
Trends of the biodegradation degree over digestion time under (**a**) mesophilic and (**b**) thermophilic conditions.

Further indications about the biodegradability of the materials can be derived from Figure 7, which shows the correlation between the biodegradation degree and the digestion time. Leaving aside the previous considerations regarding the inhomogeneity of the degradation conditions, if the acceptability criteria for anaerobic degradability of biopolymers set by the EN 13,432 (a minimum of 50% biodegradation within 60 days (red squares in Figure 7 [112])) are taken as a reference, under mesophilic conditions, most of the PHA and TPS samples, as well as some starch-based and PLA materials, would meet such criteria; on the other hand, the same types of biopolymers, along with PCL, would fulfil the same conditions in the thermophilic regime.

## 6. Conclusions

In the present paper, an updated review of the relevant findings on the biodegradability profile for typical biopolymers and related commercial bioplastics under anaerobic conditions was conducted. Particular attention was paid to expanding the current knowledge on the topic by including the results of the most recent (years 2022 and early 2023) scientific publications.

The main findings of the literature review conducted in the present work can be summarized as follows:

- The research on the topic is relatively new and has progressed considerably over the last two decades, moving from a general assessment of different biopolymers and their degradation to the evaluation of the environmental behavior of bioplastics and of the most suitable management strategies once they are discarded as wastes. It was also evident that interest in the topic has grown remarkably over the last two years, likely as a result of, among other factors, those related to the implementation of environmental policies on single-use plastic products in different countries all over the world. This testifies that the assessment of the environmental behavior of bioplastics is currently a hot topic that will deserve further attention in the years to come;
- The data extracted during the detailed analysis of the available literature (regarding the polymer characteristics, the testing conditions, the analytical techniques used to assess biodegradation, the observed biogas/methane production yield, and the estimated degree of biodegradation) indicated that the investigated bioplastics can be grouped into three main categories with regard to their response to anaerobic degradation (at least within the investigated conditions available):
  - PHAs and TPS in most cases display high levels of biodegradation regardless of the test conditions;
  - PBAT, PBS, PVA, and Mater-Bi show a low degree of conversion regardless of the temperature regime (mesophilic or thermophilic) of the degradation process;
  - PLA, PCL, and various PLA blends have a notably large variability in their biodegradation behavior, although this is observed to improve or to be less scattered when shifting to thermophilic conditions.
- At the current state of the art of biological treatment of bioplastics, the application of anaerobic digestion for the purpose of energy recovery would be feasible and economically viable for some selected types of bioplastics only. In particular, various types of PHAs, PLA, TPS, and cellulose-based polymers were found to display relatively high methane production yields, with average values between ~260 and ~380 L CH_4_/kgVS under mesophilic conditions and between ~170 and ~450 L CH_4_/kgVS under thermophilic conditions.

Additional considerations can be drawn from the analyzed data, which may be useful in outlining further critical and open issues which need to be addressed. The main questions that have arisen from the present review include the following:

- The experimental investigations were mainly carried out on pure biopolymers or ad hoc synthesized blends, while studies of commercial products are currently much more limited. Understanding the behavior of commercial bioplastic products also requires detailed knowledge of the composition of the specific blend of concern and its influence on the biodegradation features. Since the proprietary formulation of commercial blends may vary—even remarkably, depending on the intended uses of the bioplastic material—it is extremely important to relate the nature of the polymeric matrix to its biodegradation characteristics;
- While anaerobic degradation was mainly monitored through measurements of the evolved methane/biogas, additional advanced analytical techniques would be useful to describe the complex mechanisms involved in the degradation pathways;
- Harmonizing the approaches to the evaluation of bioplastic degradation and the way of expressing data is recommended to facilitate the comparison of experimental results and allow a thorough understanding of the process;
- Most of the studies have been carried out under mesophilic conditions and in a batch mode at the laboratory scale; therefore, exploring the real behavior of bioplastics at a larger scale is a matter deserving more extensive exploration. Further attention should also be paid to the effect of the degradation conditions on the kinetics and yields of the transformations involved, which may also assist in the identification of potentially useful pre-treatments that may be applied to enhance biodegradability;
- With regard to the management of bioplastic waste, in a short-to-medium-term scenario in which the collection and treatment of such residues is envisaged to be performed together with biowaste, it would be of paramount importance to assess the quality of the final digestate and its potential ecotoxicity. This would be required to identify potential environmental issues related to the presence of residual bioplastics (including microparticles).

## Data Availability

The data presented in this study are available within the article.

## Appendix A

**Table A1:** Summary of literature results related to anaerobic degradation of different bioplastic products under mesophilic conditions (expanded from [93,109]).

Class	Bioplastic Type	Size and Shape	T	Test Conditions	Time	Biogas/Methane Production						Degree of Biodegr.	Pre-Treatment	Biodegr. Eval.	Mass Loss	Analytical Techniques	Visual Insp.	Microb. Charact.	Ref.
			(°C)		(d)	^(1)^	^(2)^	^(3)^	^(4)^	^(5)^	^(6)^	(%)			(%)				
Cellulose-based	Bioceta (Cellulose acetate)	5 × 5 mm, 90 μm of thickness film	35	Plastic: 600 mg L^−1^. Inoculum: domestic sewage sludge	60	-						22 *		CH_4_ & biogas					[113]
Cellulose-based	Sugar cane cellulosic fiber plates	2 mm	37	ISR = 2 (VS basis)	250	391.1								CH_4_					[114]
Cellulose-based	Sugar cane cellulosic fiber plates	2 mm	37	ISR = 2 (VS basis)	250	342.6							48 h, acidic pretreatment (HCl) to pH = 2	CH_4_					
Cellulose-based	Sugar cane cellulosic fiber plates	2 mm	37	ISR = 2 (VS basis)	250	339.9							48 h, alkaline pretreatment (NaOH) to pH = 12	CH_4_					
Cellulose-based	Cellulose-based metallised film	1 × 1 cm film	37	Plastic to inoculum ratio: 0.25 (VS basis). Inoculum: digestate from a mesophilic digester treating municipal wastewater biosolids	65	-						74.3			88.9				[115]
Cellulose-based	Cellulose-based heat-sealable film	1 × 1 cm film	37	Plastic to inoculum ratio: 0.25 (VS basis). Inoculum: digestate from a mesophilic digester treating municipal wastewater biosolids	65	-						86.6			98.3				
Cellulose-based	Cellulose-based high barrier heat-sealable film	1 × 1 cm film	37	Plastic to inoculum ratio: 0.25 (VS basis). Inoculum: digestate from a mesophilic digester treating municipal wastewater biosolids	65	-						84			98.0				
Cellulose-based	Cellulose-based non heat-sealable film	1 × 1 cm film	37	Plastic to inoculum ratio: 0.25 (VS basis). Inoculum: digestate from a mesophilic digester treating municipal wastewater biosolids	65	-						80.4			96.4				
Cellulose-based	Cellulose diacetate film	1 × 1 cm film	37	Plastic to inoculum ratio: 0.25 (VS basis). Inoculum: digestate from a mesophilic digester treating municipal wastewater biosolids	65	-						8.9			10.3				
Cellulose-based	Cellulosic plates	Plate	35	Plastic to inoculum ratio: 0.25 (VS basis). Inoculum: digestate from a mesophilic digester treating municipal wastewater biosolids	44	311								CH_4_	100		x		[116]
Cellulose-based	Cellulosic plates	Plate	35	Plastic to inoculum ratio: 0.25 (VS basis). Inoculum: digestate from a mesophilic digester treating municipal wastewater biosolids	30	304								CH_4_	100		x		
Cellulose-based	Cellulosic plates	Plate	35	Plastic to inoculum ratio: 0.25 (VS basis). Inoculum: digestate from a mesophilic digester treating municipal wastewater biosolids	15	276								CH_4_	99.9		x		
Cellulose-based	Cellulose acetate	25 × 25 mm	37	400 g (ww) inoculum + 4.74 g (ww) CA; I/S = 2 (VS basis)	30	519.3						106		CH_4_			x		[117]
Mater-Bi	Mater-Bi (PCL + starch, Novamont)	Pieces of plastic bag < 1 mm	35	Plastic: 1 g. Inoculum: 5 mL of pig slurry mixed with synthetic medium for methanogens and acclimated to mesophilic anaerobic condition	90	33						6					x		[12]
Mater-Bi	Mater-Bi (Starch + PE, AF08H, Novamont)	2 × 15 cm strips	35	Inoculum: Mixture of sewage sludge treating domestic sewage and paper sludge (3:1 ratio)	40	-						32			53	FT-IR; NMR; UV/VIS	x		[118]
Mater-Bi	Mater-Bi (Starch + PE, AF10H, Novamont)	2 × 15 cm strips	35	Inoculum: Mixture of sewage sludge treating domestic sewage and paper sludge (3:1 ratio)	40	-						30			53	FT-IR; NMR; UV/VIS	x		
Mater-Bi	Mater-Bi (60% starch, 40% hydrophilic resin)	Whole bag	35	Plastic to inoculum ratio: 0.5 (VS basis). Inoculum: liquid digestate from an anaerobic digester fed with manure, agro-wastes and residues	15	144								CH_4_	27.5		x		[116]
Mater-Bi	Mater-Bi (60% starch, 40% hydropilic resin)	Whole bag	35	Inoculum: liquid digestate from a full-scale mesophilic digester fed with manure and agro-wastes	15	203							Alkaline pretreatment (NaOH, 5% TS), 24 h	CH_4_	78.2		x		
Mater-Bi	Mater-Bi (60% starch, 40% hydropilic resin)	Shredded bag (1 × 1 cm)	35	Inoculum: liquid digestate from a full-scale mesophilic digester fed with manure and agro-wastes	15	117							Mechanical shredding	CH_4_	29.3		x		
Mater-Bi	Mater-Bi (60% starch, 40% hydropilic resin)	Pre-digested bag (1 × 1 cm)	35	Inoculum: liquid digestate from a full-scale mesophilic digester fed with manure and agro-wastes	15	33							Pre-digestion treatment (mesophilic)	CH_4_	4.8		x		
Mater-Bi	Mater-Bi (60% starch, 40% hydropilic resin)	Pre-digested bag (1 × 1 cm)	35	Inoculum: liquid digestate from a full-scale mesophilic digester fed with manure and agro-wastes	15	27							Alkaline pre-treatment (NaOH, 5% TS, 24 h) on pre-digested (mesophilic) samples	CH_4_	−0.3		x		
Mater-Bi	Mater-Bi (60% starch, 40% hydropilic resin)	Whole bag	35	Inoculum: liquid digestate from a full-scale mesophilic digester fed with manure and agro-wastes, pre-acclimated	15	42								CH_4_			x		
Mater-Bi	Mater-Bi (60% starch, 40% hydropilic resin)	Pre-digested bag (1 × 1 cm)	35	Inoculum: liquid digestate from a full-scale mesophilic digester fed with manure and agro-wastes, pre-acclimated	15	66							Pre-digestion treatment (mesophilic)	CH_4_			x		
Mater-Bi	Mater-Bi (60% starch, 40% hydropilic resin)	Pre-digested bag (1 × 1 cm)	35	Inoculum: liquid digestate from a full-scale mesophilic digester fed with manure and agro-wastes, pre-acclimated	15	70							Alkaline pre-treatment (NaOH, 5% TS, 24 h) on pre-digested (mesophilic) samples	CH_4_			x		
Mater-Bi	Mater-Bi (PCL+Starch+Glycerin, ZI01U, Novamont)	Film	35	Inoculum: anaerobic sludge from an anaerobic digester. Method: ASTM D 5511-94	81	203.6						21		X		TGA, SEM			[119]
Mater-Bi	Mater-Bi (PCL+Starch+Glycerin, ZI01U, Novamont)	Pellets	35	Inoculum: anaerobic sludge from an anaerobic digester. Method: ASTM D 5511-94	81	96.4						10		X		SEM			
Mater-Bi	Mater-Bi (Starch + PCL, Novamont)	2 × 2 cm film 20 μm of thickness	35		28		485.2					23		X	44.8	FTIR, SEC, NMR, DSC	X		[70]
Mater-Bi	Mater-Bi ZF03U (PCL + starch, Novamont)	5 × 5 mm 35 μm of thickness	35	Plastic: 600 and 400 mg L^−1^. Inoculum: domestic sewage sludge	60							28		CH_4_ & biogas					[113]
Mater-Bi	Mater-Bi (Novamont)	0.5–1 mm film	35	Plastic to inoculum ratio: 0.6–1 (TS basis). Inoculum: anaerobic sludge from an anaerobic digestion plant treating effluents from a brewery Method: ASTM D5526-94d.	32	220													[120]
Mater-Bi	Mater-Bi bags	10 × 10 mm film	37	Inoculum: anaerobic sludge from an anaerobic digestion plant treating municipal wastewater	180		30.4					2.9		X		FTIR, DSC, microscopy	x		[121]
Mater-Bi	Mater-Bi coffee capsules	<1 mm	38	Inoculum: sludge from a wastewater treatment plant, acclimated in the lab at 38 °C. Digestion conditions: ISR = 2.7 (VS basis), VS content = 9 g/L	100	67						12		X				x	[95]
PBAT	PBAT	2 × 2 cm film 20 μm of thickness	35		28							0		X	44.8	FTIR, SEC, NMR, DSC	x		[70]
PBAT	PBAT 93,000 g/mol (Ecoflex, BASF)	5 × 5 mm film 70 μm of thickness	37	Inoculum: mesophilic anaerobic sludge (37 °C) from a municipal waste water-treatment plant	126							2.2 *		X	2.8	DSC, XRD, GPC			[122]
PBAT	PBAT	1 mm sheet	38	I/S = 2.85 (VS basis); working V = 300 mL	500	159.7						13.4		CH_4_				x	[95]
PBAT		0.1–0.25 mm	36	Anaerobic aqueous conditions ISO 14853; working V = 1 L; 1 gTS/L inoculum + 150 mg/L test material	77							0		Biogas					[123]
PBS	PBES (MW 100,000, Sky Green)	20 × 40 mm film	35	Inoculum: anaerobic digested sludge from a WWTP. Method: ASTM D5210	100							0		X	35				[124]
PBS	PBS (Sigma-Aldrich)	125–250 μm	37	Plastic: 10 g. Inoculum: mesophilic digestate from a mesophilic anaerobic digester treating cow manure and green waste	277							0 *		X				x	[100]
PBS	PBS (Elson Green)	20 × 40 mm film	35	Inoculum: anaerobic digested sludge from a WWTP. Method: ASTM D5210	100							0		X	28				[124]
PBS	PBS		35	Method: ASTM E1196-92	100	11						2		CH_4_ & biogas					[125]
PBS	PBS (PBE 003, NaturePlast,)	<2 × 2 cm	35	Inoculum: sludge from a WWTP. Method: ISO 14853	56							0		biogas		SEM			[126]
PBS	PBS (Enpol G4560, IRE Chemical Ltd.)	5 × 5 mm thin film (100 μm thickness)	37	Plastic: 100 mg. Inoculum: mesophilic anaerobic sludge from a wastewater treatment plant. Method: ISO 11734	113							2.2		biogas		DSC, XRD, SEM			[127]
PBS	PBS	1 mm sheet	38	I/S = 2.85 (VS basis); working volume = 300 mL	500	0						0		CH_4_				x	[95]
PBS		0.1–0.25 mm	36	Anaerobic aqueous conditions ISO 14853; working V = 1 L; 1 gTS/L inoculum + 150 mg/L test material	77							3.1		Biogas					[123]
PCL	PCL (Sigma-Aldrich)	125–250 μm	37		277							3		X					[100]
PCL	PCL (Sigma-Aldrich)	125–250 μm	37		277							22		X					
PCL	PCL (MW 50,000 g.mol^−1^, Polyscience Inc.)	27 mm of diameter 100 μm of thickness film	39	Plastic: 0.2 g. Inoculum: sludge from a laboratory anaerobic reactor treating wastewater from a sugar factory. Method: ASTM D 5210-93	42							7.5 *		X	30				[97]
PCL	PCL (MW 50,000 g.mol^−1^, Polyscience Inc.)	19 mm of diameter film	37	Plastic: 35–40 mg. Inoculum: sludge from an anaerobic laboratory reactor fed with wastewater from sugar industry. Method: ASTM D 5210-91	42							16		Biogas	30			x	[128]
PCL	1,4-butanediol/adipic acid (MW 40,000, GBF)	19 mm of diameter film	37	Plastic: 35–40 mg. Inoculum: sludge from an anaerobic laboratory reactor fed with wastewater from sugar industry. Method: ASTM D 5210-91	42							1.1		Biogas	1.2			x	
PCL	1,4-butanediol (50 mol%) adipic acid (30 mol%)/Terephthalic acid (20 mol%) (MW 47,600, Hüls AG)	19 mm of diameter film	37	Plastic: 35–40 mg. Inoculum: sludge from an anaerobic laboratory reactor fed with wastewater from sugar industry. Method: ASTM D 5210-91	42							5.5		Biogas	0.5			x	
PCL	PCL (MW 50,000 g.mol^−1^, Polyscience Inc.)	19 mm of diameter film	37	Plastic: 35–40 mg. Inoculum: sludge from an anaerobic digester of a municipal WWTP. Method: ASTM D 5210-91	42							17		Biogas	30			x	
PCL	1,4-butanediol/adipic acid (MW 40,000, GBF)	19 mm of diameter film	37	Plastic: 35–40 mg. Inoculum: sludge from an anaerobic digester of a municipal WWTP. Method: ASTM D 5210-91	42							11		Biogas	2.1			x	
PCL	1,4-butanediol (50 mol%) adipic acid (30 mol%)/Terephthalic acid (20 mol%)	19 mm of diameter film	37	Plastic: 35–40 mg. Inoculum: sludge from an anaerobic digester of a municipal WWTP. Method: ASTM D 5210-91	42							11		Biogas	1%			x	
PCL	PCL		35	Plastic: 10 mg.L^−1^. Inoculum: digestate from an anaerobic digester treating WWTP sludge	122							0.2		CH_4_ and biogas					[129]
PCL	PCL	1 cm^2^ film pieces	37	Plastic to inoculum ratio: 0.5 (VS basis). Inoculum: digestate from a mesophilic anaerobic digester fed with food waste and manure	30	15.8						6.5		CH_4_					[130]
PCL	PCL 40% TPS 60%	1 cm^2^ film pieces	37	Plastic to inoculum ratio: 0.5 (VS basis). Inoculum: digestate from a mesophilic anaerobic digester fed with food waste and manure	30	133.3						32.3		CH_4_					
PCL	PCL 60% TPS 40%	1 cm^2^ film pieces	37	Plastic to inoculum ratio: 0.5 (VS basis). Inoculum: digestate from a mesophilic anaerobic digester fed with food waste and manure	30	74.2						18.5		CH_4_					
PCL	PCL (Tone, Union Carbide)	2 × 15 cm strips	35	Inoculum: Mixture of sewage sludge treating domestic sewage and paper sludge (3:1 ratio)	40							5			6%	FTIR, NMR, UV/VIS, SEM			[118]
PCL	Ecostarplus (starch + PE)	2 × 15 cm strips	35	Inoculum: Mixture of sewage sludge treating domestic sewage and paper sludge (3:1 ratio)	40							12			5%	FTIR; NMR; UV/VIS; SEM			
PCL	PCL (Tone, Union Carbide)	Powder	35	Inoculum: 2 mL of digestate from an anaerobic digester treating sewage sludge. Method: ISO 14853	28							0		X	0%	FTIR, SEC, NMR, DSC, SEM			[70]
PCL	PCL (CAPA 6500, Perstorp)	<2 × 2 cm	35	Inoculum: sludge from a WWTP. Method: ISO 14853	56							3		Biogas		DSC, SEM			[126]
PCL	PCL (P787, Union Carbide)	5 × 5 mm 55 μm of thickness and 250 μm powder	35	Plastic: 600 and 400 mg/L. Inoculum: domestic sewage sludge	60							0		CH_4_ & biogas					[113]
PCL	PCL	1 mm sheet	38	I/S = 2.85 (VS basis); working volume = 300 mL	500	366.9						49.9		CH_4_				x	[95]
PCL		0.1–0.25 mm	36	Anaerobic aqueous conditions ISO 14853; working V = 1 L; 1 g TS/L inoculum + 150 mg/L test material	77							4.5		Biogas					[123]
PCL	film	0.25 × 0.25 cm	35	ASTM D 5210-91; 150 mL working V + 100 mg polymer; flushed with N_2_	77							0		Biogas					[131]
PCL	film	0.25 × 0.25 cm	35	ISO 11734; 150 mL working V + 100 mg polymer; flushed with N_2_	77							1		Biogas					
PCL	powder		35		58.3							2		Biogas	6.5	TGA, DSC, SEM			[132]
PCL *	PCL-Starch blend (55% PCL, 30% Starch, 15% aliphatic polyester)		35	Plastic to inoculum ratio: 2 g VS/L, Inoculum: 20 mL digestate from a anaerobic digester treating sewage sludge.	139	554						83		CH_4_ & biogas					[125]
PCL+PHO	PCL/PHO (85/15)	<2 × 2 cm	35	Inoculum: sludge from a WWTP. Method: ISO 14853	56							4		Biogas		DSC, SEM			[126]
PCL+TPS	PCL/TPS (70/30)	<2 × 2 cm	35	Inoculum: sludge from a WWTP. Method: ISO 14853	56							36		Biogas		DSC, SEM			
PCL61/S-A26/G13	PCL+starch+glycerol	50 × 9(4) × 1 mm	35		58.3							30.3		Biogas	30.6	TGA, DSC, SEM, mech. properties		x	[132]
PCL61/S-GI26/G13	PCL+starch+glycerol	50 × 9(4) × 1 mm	35		58.3							29.8		Biogas	30.4	TGA, DSC, SEM			
PCL61/S-M26/G13	PCL+starch+glycerol	50 × 9(4) × 1 mm	35		58.3							12.6		Biogas	13.8	TGA, DSC, SEM			
PCL61/S-W26/G13	PCL+starch+glycerol	50 × 9(4) × 1 mm	35		58.3							31.2		Biogas	30.7	TGA, DSC, SEM			
PCL70/S-A30	PCL+starch	50 × 9(4) × 1 mm	35		58.3							10.1		Biogas	11.9	TGA, DSC, SEM			
PCL70/S-GI30	PCL+starch	50 × 9(4) × 1 mm	35		58.3							10.4		Biogas	13.9	TGA, DSC, SEM			
PCL70/S-M30	PCL+starch	50 × 9(4) × 1 mm	35		58.3							5.6		Biogas	6.5	TGA, DSC, SEM			
PCL70/S-W30	PCL+starch	50 × 9(4) × 1 mm	35		58.3							10.7		Biogas	9.8	TGA, DSC, SEM			
PHA	PHA (PHA-4100, Metabolix)	1–2 mm wide pellets	37	Plastic to inoculum ratio: 4 g/L. Inoculum: sludge from a semi continuous anaerobic digester fed with food waste, olive, and cheese waste. Method: ASTM 5511-02	11							102		Biogas					[133]
PHA	PHA (PHA-4100, Metabolix)	1–2 mm wide pellets	37	Plastic to inoculum ratio: 8 g/L. Inoculum: sludge from a semi continuous anaerobic digester fed with food waste, olive, and cheese waste. Method: ASTM 5511-02	11							95		Biogas					
PHA	PHA	PHA accumulated in activated sludge	37	Plastic: addition of 1 mL of PHA-accumulating sludge (30 g TS/L). Inoculum: 5 mL of sewage sludge from a WWTP	15	250						53		Biogas					[134]
PHB	PHB (ENMAT Y3000, TianAn)	<0.15 mm	35	Plastic: 125 mg. Inoculum: 50 mL of lab inoculum fed with nutritive media and powdered milk	40					199		50		CH_4_					[11]
PHB	PHB (ENMAT)	<0.15 mm	35	Plastic: 125 mg. Inoculum: 50 mL of lab inoculum fed with nutritive media and powdered milk	40					398		100	35 °C, addition of NaOH until pH 12 for 24 h	CH_4_					
PHB	PHB (MIREL F1006, Metabolix)	<0.15 mm	35	Plastic: 125 mg. Inoculum: 50 mL of lab inoculum fed with nutritive media and powdered milk	40					233		59		CH_4_					
PHB	PHB (Mirel F1006)	<0.15 mm	35	Plastic: 125 mg. Inoculum: 50 mL of lab inoculum fed with nutritive media and powdered milk	40					359		90.9	35 °C, pH 7 for 48 h	CH_4_					
PHB	PHB (Mango materials)	<0.15 mm	35	Plastic: 125 mg. Inoculum: 50 mL of lab inoculum fed with nutritive media and powdered milk	40					316		80		CH_4_					
PHB	PHB (Mango materials)	<0.15 mm	35	Plastic: 125 mg. Inoculum: 50 mL of lab inoculum fed with nutritive media and powdered milk	40					322		81.5	55 °C, addition of NaOH until pH = 10, 24 h	CH_4_					
PHB	PHB (Mirel M2100, Metabolix)	<0.15 mm	35	Plastic: 125 mg. Inoculum: 50 mL of lab inoculum fed with nutritive media and powdered milk	40					316		80		CH_4_					
PHB	PHB (Mirel M2100, Metabolix)	<0.15 mm	35	Plastic: 125 mg. Inoculum: 50 mL of lab inoculum fed with nutritive media and powdered milk	40					357		90.4	55 °C, addition of NaOH until pH = 12, 24 h	CH_4_					
PHB	PHB (Sigma-Aldrich)	125–250 μm	37		9							90		X					[100]
PHB	PHB (MW 540,000 g.mol^−1^, Biopol BX G08)	25 mm of diameter 100 μm of thickness film	37	Plastic: 0.2 g. Inoculum: sludge from a laboratory anaerobic reactor treating wastewater from a sugar factory. Method: ASTM D 5210-91	9							100		Biogas	100				[97]
PHB	PHB (MW 540,000 g.mol^−1^, Biopol BX G08)	19 mm of diameter film	37	Plastic: 35–40 mg. Inoculum: sludge from an anaerobic laboratory reactor fed with wastewater from sugar industry. Method: ASTM D 5210-91	8							101		Biogas					[128]
PHB	PHB (MW 540,000 g.mol^−1^, Biopol BX G08)	19 mm of diameter film	37	Plastic: 35–40 mg. Inoculum: sludge from an anaerobic laboratory reactor fed with wastewater from sugar industry. Method: ASTM D 5210-92	42							101		Biogas	100				
PHB	PHB (MW 540,000 g.mol^−1^, Biopol BX G08)	19 mm of diameter film	37	Plastic: 35–40 mg. Inoculum: sludge from an anaerobic digester of a municipal WWTP. Method: ASTM D 5210-91	8							100		Biogas					
PHB	PHB (MW 540,000 g.mol^−1^, Biopol BX G08)	19 mm of diameter film	37	Plastic: 35–40 mg. Inoculum: sludge from an anaerobic digester of a municipal WWTP. Method: ASTM D 5210-91	42							101		Biogas	100				
PHB	PHB	Granular form	35	Plastic to inoculum ratio: 10 g VS g^−1^ VS. Inoculum: digestate from a WWTP anaerobic digester.	23							100							[135]
PHB	PHB	Powder	35	Plastic: 5 mg. Inoculum: anaerobically digested domestic sewage sludge	16							87		Biogas					[136]
PHB	PHB (ENMAT Y1000, TianAn)	<2 × 2 cm	35	Inoculum: sludge from a WWTP. Method: ISO 14853	56	-						102		Biogas		DSC, SEM			[126]
PHB	PHB (MW 539,000, Biopol BX G08)	200 μm powder	35	Plastic: 400 mg L^−1^. Inoculum: domestic sewage sludge	30	-						80		CH_4_ & biogas					[113]
PHB	PHB Biomer	1 mm sheet	38	I/S = 2.85 (VS basis); working volume = 300 mL	50	383.4						64.3		CH_4_				x	[95]
PHB	PHB (K. D.)	1 mm sheet	38	I/S = 2.85 (VS basis); working volume = 300 mL	25	491.5						80.1		CH_4_				x	
PHB	PHB (K.D.)	particles 1.01 mm (mean size)	38	I/S = 10 (VS basis)	23	518						94		CH_4_					[99]
PHB	PHB (K.D.)	particles 1.01 mm (mean size)	38	I/S = 4 (VS basis)	23	483						88		CH_4_					
PHB	PHB (K.D.)	particles 1.01 mm (mean size)	38	I/S = 2.85 (VS basis)	18	518						94		CH_4_					
PHB	PHB (K.D.)	particles 1.01 mm (mean size)	38	I/S = 2 (VS basis)	38	468						85		CH_4_					
PHB	PHB (K.D.)	particles 1.01 mm (mean size)	38	I/S = 1 (VS basis)	15	51						9		CH_4_					
PHB		0.1–0.25 mm	36	Anaerobic aqueous conditions ISO 14853; working V = 1 L; 1 g TS/L inoculum + 150 mg/L test material	77							83.9		Biogas					[123]
PHB		0.1–0.25 mm	36	Anaerobic standard test conditions—ISO 14852; polymer = 1 g VS/L	77			495.8				85		Biogas					
PHB		0.1–0.25 mm	36	Anaerobic standard test conditions—ISO 14852; polymer = 1 g VS/L	100				815.7			78.4		Biogas					
PHB		0.25–0.5 mm	36	Anaerobic standard test conditions—ISO 14852; polymer = 1 g VS/L	100				759.3			72.9		Biogas					
PHB		0.5–1 mm	36	Anaerobic standard test conditions—ISO 14852; polymer = 1 g VS/L	100				648.9			62.3		Biogas					
PHB	Plates	1.1 × 4.5 × 1.2 mm	35	Working V = 150 mL; polymer = 8 mg C/L	85				1364			73.0		Biogas	100	TGA, DSC, SEM			[137]
PHB	Plates	1.1 × 4.5 × 1.2 mm	35	Working V = 150 mL; polymer = 4.225 mg C/L	65				1253			67.0		Biogas		TGA, DSC, SEM			
PHB	Plates	1.1 × 4.5 × 1.2 mm	35	Working V = 150 mL; polymer = 4.665 mg C/L	80				1546			82.8		Biogas	79.1	TGA, DSC, SEM			
PHB powder			35	Working V = 150 mL; polymer = 1 mg C/L					1185			63.4		Biogas		TGA, DSC, SEM			
PHB powder			35	Working V = 150 mL; polymer = 1 mg C/L					1274			68.0		Biogas		TGA, DSC, SEM			
PHB/PHV	Film 0.06 mm	0.2–0.63 mm	35	ASTM D 5210-91; 150 mL working V + 100 mg polymer; flushed with N_2_	41							70		Biogas					[131]
PHB/PHV	Film 0.06 mm	0.2–0.63 mm	35	ASTM D 5210-91; 150 mL working V + 100 mg polymer; flushed with 70% N_2_/30% CO_2_	33							64		Biogas					
PHB/PHV	Film 0.06 mm	0.2–0.63 mm	35	ISO 11734; 150 mL working V + 100 mg polymer; flushed with N_2_	41							62		Biogas					
PHB/PHV	Film 0.06 mm	0.2–0.63 mm	35	ISO 11734; 150 mL working V + 100 mg polymer; flushed with 70% N_2_/30% CO_2_	33							64		Biogas					
PHB/TBC (85/15)	Plates; TBC = tributyl citrate	1.1 × 4.5 × 1.2 mm	35	Working V = 150 mL; polymer = 4.004 mg C/L	190							93.8		Biogas		FTIR, DSC, SEM			[137]
PHB+PBS	PHB/PBS (50/50)	<2 × 2 cm	35	Inoculum: sludge from a WWTP. Method: ISO 14853	56	-						15		Biogas		DSC, SEM			[126]
PHB+PCL	PHB/PCL (60/40)	<2 × 2 cm	35	Inoculum: sludge from a WWTP. Method: ISO 14853	56	-						38		Biogas		DSC, SEM			
PHB+PHH	Poly(3-hydroxybutyrate-co-3-hydroxyhexanoate) 93% HB, 7% HHx	5 × 5 × 1 mm Film	38	Plastic to inoculum ratio: 0.7–0.8 (VS basis). Inoculum: Digestate from a mesophilic anaerobic digester fed with sludge and fats	80	483.8						77				GPC		x	[138]
PHB+PHH	Poly(3-hydroxybutyrate-co-3-hydroxyhexanoate) 93.5% HB 6.5% HHx	5 × 5 × 1 mm Flake	38	Plastic to inoculum ratio: 0.7–0.8 (VS basis). Inoculum: Digestate from a mesophilic anaerobic digester fed with sludge and fats	40	337.5						54						x	
PHB+PHH	Poly(3-hydroxybutyrate-co-3-hydroxyhexanoate) 93.5% HB 6.5% HHx	5 × 5 × 1 mm Flake	38	Plastic to inoculum ratio: 0.7–0.8 (VS basis). Inoculum: Digestate from a mesophilic anaerobic digester fed with sludge and fats	80	337.5						54			51.9				
PHB+PHO	PHB/PHO (85/15)	<2 × 2 cm	35	Inoculum: sludge from a WWTP. Method: ISO 14853	56	-						92		Biogas		DSC, SEM			[126]
PHBO	PHBO (90% PHB, 10% HO)		35	Plastic: 100 mg/L. Inoculum: digestate from an anaerobic digester treating WWTP sludge.	60	-						88		CH_4_ & biogas					[129]
PHBV	PHBV (0.5% HV, ENMAT Y1000P)	31.25 mm × 6.2 mm × 2.1 mm rectangular prism	37	Plastic to inoculum ratio: 0.5 (VS basis). Inoculum: digestate from a mesophilic digester.	42			630				83		CH_4_		SEM, 3D imaging with µCT			[139]
PHBV	PHBV (ENMAT Y1000P China)	Rectangular prism 31.25 mm × 6.2 mm × 2.1 mm	37	Neat PHBV	80							94		CH_4_	100	SEM, 3D imaging with µCT			
PHBV	Maleated PHBV	Rectangular prism 31.25 mm × 6.2 mm × 2.1 mm	37	Maleated PHBV	80							95		CH_4_	100	SEM, 3D imaging with µCT			
PHBV	PHBV (0.5% HV ENMAT Y1000P)	420–840 μm	37	Plastic to inoculum ratio: 0.5 (VS basis). Inoculum: digestate from a mesophilic digester treating municipal wastewater	20			580				86		CH_4_					[140]
PHBV	PHBV (0.5% HV ENMAT Y1000P)	3900 μm (pellets)	37	Plastic to inoculum ratio: 0.5 (VS basis). Inoculum: digestate from a mesophilic digester treating municipal wastewater	36			580				86	Size reduction	CH_4_					
PHBV	PHBV (0.5% HV ENMAT Y1000P)	420–840 μm	37	Plastic to inoculum ratio: 0.5 (VS basis). Inoculum: digestate from a mesophilic digester treating municipal wastewater	20			580				86	Size reduction	CH_4_					
PHBV	PHBV (0.5% HV ENMAT Y1000P)	250–420 μm	37	Plastic to inoculum ratio: 0.5 (VS basis). Inoculum: digestate from a mesophilic digester treating municipal wastewater	22			580				86	Size reduction	CH_4_					
PHBV	PHBV (0.5% HV ENMAT Y1000P)	150–250 μm	37	Plastic to inoculum ratio: 0.5 (VS basis). Inoculum: digestate from a mesophilic digester treating municipal wastewater	19			580				86	Size reduction	CH_4_					
PHBV	PHBV (0.5% HV ENMAT Y1000P)	10 μm	37	Plastic to inoculum ratio: 0.5 (VS basis). Inoculum: digestate from a mesophilic digester treating municipal wastewater	23			580				86	Size reduction	CH_4_					
PHBV	PHBV (0.5% HV ENMAT Y1000P)	Rectangular prism 31.25 mm × 6.2 mm × 2.1 mm	37		42			630				83		CH_4_	38	DSC			[66]
PHBV	PHBV (MW 397,000 g.mol^−1^, Biopol BX P027)	26 mm of diameter 100 μm of thickness film	38	Plastic: 0.2 g. Inoculum: sludge from a laboratory anaerobic reactor treating wastewater from a sugar factory. Method: ASTM D 5210-92	42							29		Biogas	60				[97]
PHBV	PHBV (MW 397,000 g.mol^−1^, Biopol BX P027)	19 mm of diameter film	37	Plastic: 35–40 mg. Inoculum: sludge from an anaerobic laboratory reactor fed with wastewater from sugar industry. Method: ASTM D 5210-91	42							29		Biogas	57				[128]
PHBV	PHBV (MW 397,000 g.mol^−1^, Biopol BX P027)	19 mm of diameter film	37	Plastic: 35–40 mg. Inoculum: sludge from an anaerobic digester of a municipal WWTP. Method: ASTM D 5210-91	42							31		Biogas	63				
PHBV	PHBV (PHB/HV; 92/8, *w*/*w*)	5 × 60 mm film	35	Inoculum: anaerobic digested sludge from a WWTP. Method: ASTM D5210	20							85		Biogas					[124]
PHBV	Cellophane	20 × 40 mm film	35	Inoculum: anaerobic digested sludge from a WWTP. Method: ASTM D5210	20							80		Biogas					
PHBV	PHBV (ICI)	2 × 15 cm strips	35	Inoculum: Mixture of sewage sludge treating domestic sewage and paper sludge (3:1 ratio)	40							55			29	FT-IR; NMR; UV/VIS	x		[118]
PHBV	PHBV (13% HV)	Powder	35	Plastic: 5 mg. Inoculum: anaerobically digested domestic sewage sludge	16							96		Biogas					[136]
PHBV	PHBV (20% HV)	Powder	35	Plastic: 5 mg. Inoculum: anaerobically digested domestic sewage sludge	16							83		Biogas					
PHBV	PHBV (8.4% HV, ICI)	46.4 μm	35	Plastic: 1% *w*/*w*, Inoculum: 10% *w*/*w* anaerobic sludge from a WWTP of a sugar factory	30							95		Biogas					[141]
PHBV	PHBV	Pellets	35	Inoculum: 1:1 mixture of mesophilic and thermophilic digestate from lab-scale AD reactors. ISR = 1 (VS basis). Solids content in the reactor: 7.22% TS	104	271										SEM			[142]
PHBV		0.1–0.25 mm	36	Anaerobic aqueous conditions ISO 14853; working V = 1 L; 1 g TS/L inoculum + 150 mg/L test material	77							81.2		Biogas					[123]
PHBV		0.1–0.25 mm	36	Anaerobic standard test conditions—ISO 14852; polymer = 1 g VS/L	77			480.1				76.4		Biogas					
PHBV		0.1–0.25 mm	36	Anaerobic standard test conditions—ISO 14852; polymer = 1 g VS/L	100				792.3			73.2		Biogas					
PHBV		0.25–0.5 mm	36	Anaerobic standard test conditions—ISO 14852; polymer = 1 g VS/L	100				777.8			71.8		Biogas					
PHBV		0.5–1 mm	36	Anaerobic standard test conditions —ISO 14852; polymer = 1 g VS/L	100				748.8			69.1		Biogas					
PHBV+wood flour	80% PHBV 20% oak wood flour	Rectangular prism 31.25 mm × 6.2 mm × 2.1 mm	37	Addition of 20% oak wood flour	50–63							84		CH_4_	100	SEM, 3D imaging with µCT			[139]
PHBV+wood flour	80% maleated PHBV 20% oak wood flour	Rectangular prism 31.25 mm × 6.2 mm × 2.1 mm	37	Maleated PHBV + addition of oak wood flour	50–63							88		CH_4_	100	SEM, 3D imaging with µCT			
PHBV+wood flour	80% PHBV 20% silane treated oak wood flour	Rectangular prism 31.25 mm × 6.2 mm × 2.1 mm	37	Addition of silane treated oak wood flour	50–63							83		CH_4_	100	SEM, 3D imaging with µCT			
PHBV+wood flour	80% PHBV and 20% oak wood flour	Rectangular prism 31.25 mm × 6.2 mm × 2.1 mm	37	Addition of 20% oak wood flour	28				510			73		CH_4_		DSC			[66]
PHBV+wood flour	60% PHBV and 40% oak wood flour	Rectangular prism 31.25 mm × 6.2 mm × 2.1 mm	37	Addition of 40% oak wood flour	28				430			60		CH_4_		DSC			
PHO	PHO (Bioplastech R, Bioplastech)	<2 × 2 cm	35	Inoculum: sludge from a WWTP. Method: ISO 14853	56							12		Biogas		DSC, SEM			[126]
PLA	PLA (Ingeo)	Pieces of plastic cup < 1 mm	35	Plastic: 1 g. Inoculum: 5 mL of pig slurry mixed with synthetic medium for methanogens and acclimated to mesophilic anaerobic condition	90	0						0		---	0				[12]
PLA	PLA (Fabri-Kal)	Plastic cup ground to 3 mm	37	Plastic: 1 g. Inoculum: 10 mL of anaerobic inoculum	60	2						0.4							[143]
PLA	PLA (Fabri-Kal)	Plastic cup ground to 3 mm	37	Plastic: 1 g. Inoculum: 10 mL of anaerobic inoculum	56	90						19.30	Steam exposition, 3 h 120 °C						
PLA	PLA (Ingeo 2003D, NatureWorks)	0.15 mm	35	Plastic: 125 mg. Inoculum: 50 mL of lab inoculum fed with nutritive media and powdered milk	40					1		0		CH_4_					[11]
PLA	PLA (Ingeo 2003D NatureWorks)	0.15 mm	35	Plastic: 125 mg. Inoculum: 50 mL of lab inoculum fed with nutritive media and powdered milk	40					86		23.9	90 °C, addition of NaOH until pH = 10, 48 h	CH_4_					
PLA	PLA (Unitika)	125–250 μm	37		277							29		X					[100]
PLA	PLA (Unitika)	125–250 μm	37		277							49		X					
PLA	PLA (NatureWorks)	1–2 mm wide pellets	37	Plastic to inoculum ratio: 4 g/L. Inoculum: sludge from a semi continuous anaerobic digester fed with food waste, olive, and cheese waste. Method: ASTM 5511-02	20							5							[133]
PLA	PLA (lab)	20 × 40 mm film	35	Inoculum: anaerobic digested sludge from a WWTP. Method: ASTM D5210	100							0							[124]
PLA	PLA (Argonne A)	6 × 5 cm film	35	Inoculum: Mixture of sewage sludge treating domestic sewage and paper sludge (3:1 ratio)	40							10			9	FT-IR; NMR; UV/VIS	X		[118]
PLA	PLA (Argonne B)	6 × 5 cm film	35	Inoculum: Mixture of sewage sludge treating domestic sewage and paper sludge (3:1 ratio)	40							15			3	FT-IR; NMR; UV/VIS	X		
PLA	PLA	Granules	37	Plastic: 30 mg. Inoculum: anaerobic sludge from a WWTP. Method: ASTM D 5210	100							60		Biogas					[144]
PLA	PLA (NatureWorks, Cargill)	2 × 2 cm film 20 μm of thickness	35		28							0		X	0	FTIR, SEC, NMR, DSC	X		[70]
PLA	PLA (Biopolymer-4043D, NatureWorks)	<2 × 2 cm	35	Inoculum: sludge from a WWTP. Method: ISO 14853	56							0		Biogas		DSC, SEM			[126]
PLA	PLA film	1 × 1 cm film	37	Plastic to inoculum ratio: 0.25 (VS basis). Inoculum: digestate from a mesophilic digester treating municipal wastewater sludge	65							18.8			20.2				[115]
PLA	PLA blend	Pellets	37	Plastic to inoculum ratio: 0.25 (VS basis). Inoculum: digestate from a mesophilic digester treating municipal wastewater sludge	65							2.6			3.0				
PLA	PLA (plastic cup)	2 × 2 × 0.5 mm	37	Plastic to inoculum ratio: 2–4 kg VS/m^3^. Inoculum: mesophilic digestate from a mesophilic wastewater treatment plant digester. Method: EN ISO 11734:2003	280		564					66		Biogas		FTIR, opt. microscopy			[145]
PLA	Mixture of PLA goods (dishes, glasses and cutlery)	5 × 5 cm	37	Mesophilic digestate from a full-scale dry anaerobic digester treating OFMSW	60			34						CH_4_					[10]
PLA	Mixture of PLA goods (dishes, glasses and cutlery)	5 × 5 cm	37	Mesophilic digestate from a full-scale dry anaerobic digester treating OFMSW	90									CH_4_	24	FTIR			
PLA	Commercial PLA blend (80% PLA, 20% additives)	<2 mm	37	Mesophilic digestate from a full-scale dry anaerobic digester treating OFMSW	146	50.5						10.8		CH_4_					[146]
PLA	Commercial PLA blend (80% PLA, 20% additives)	<2 mm	37	Mesophilic digestate from a full-scale dry anaerobic digester treating OFMSW	40	61.3						13.1	Hydrothermal (1 g VS-PLA, T = 120 °C, 10 min, 10 mL water)	CH_4_					
PLA	Commercial PLA blend (80% PLA, 20% additives)	<2 mm	37	Mesophilic digestate from a full-scale dry anaerobic digester treating OFMSW	40	111.5						23.8	Hydrothermal (1 g VS-PLA, T = 120 °C, 30 min, 10 mL 1% NaOH)	CH_4_					
PLA	Commercial PLA blend (80% PLA, 20% additives)	<2 mm	37	Mesophilic digestate from a full-scale dry anaerobic digester treating OFMSW	40	136.1						29.1	Hydrothermal (1 g VS-PLA, T = 120 °C, 60 min, 10 mL 5% NaOH)	CH_4_					
PLA	Commercial PLA blend (80% PLA, 20% additives)	<2 mm	37	Mesophilic digestate from a full-scale dry anaerobic digester treating OFMSW	40	249.9						53.4	Hydrothermal (1 g VS-PLA, T = 120 °C, 120 min, 10 mL 10% NaOH)	CH_4_					
PLA	Commercial PLA blend (80% PLA, 20% additives)	<2 mm	37	Mesophilic digestate from a full-scale dry anaerobic digester treating OFMSW	40	161.3						34.5	Hydrothermal (1 g VS-PLA, T = 160 °C, 10 min, 10 mL 1% NaOH)	CH_4_					
PLA	Commercial PLA blend (80% PLA, 20% additives)	<2 mm	37	Mesophilic digestate from a full-scale dry anaerobic digester treating OFMSW	40	262.8						56.2	Hydrothermal (1 g VS-PLA, T = 160 °C, 30 min, 10 mL water)	CH_4_					
PLA	Commercial PLA blend (80% PLA, 20% additives)	<2 mm	37	Mesophilic digestate from a full-scale dry anaerobic digester treating OFMSW	40	432.3						92.4	Hydrothermal (1 g VS-PLA, T = 160 °C, 60 min, 10 mL 10% NaOH)	CH_4_					
PLA	Commercial PLA blend (80% PLA, 20% additives)	<2 mm	37	Mesophilic digestate from a full-scale dry anaerobic digester treating OFMSW	40	430.8						92.1	Hydrothermal (1 g VS-PLA, T = 160 °C, 120 min, 10 mL 5% NaOH)	CH_4_					
PLA	Commercial PLA blend (80% PLA, 20% additives)	<2 mm	37	Mesophilic digestate from a full-scale dry anaerobic digester treating OFMSW	40	441.6						94.4	Hydrothermal (1 g VS-PLA, T = 200 °C, 10 min, 10 mL 5% NaOH)	CH_4_					
PLA	Commercial PLA blend (80% PLA, 20% additives)	<2 mm	37	Mesophilic digestate from a full-scale dry anaerobic digester treating OFMSW	40	456						97.5	Hydrothermal (1 g VS-PLA, T = 200 °C, 30 min, 10 mL 10% NaOH)	CH_4_					
PLA	Commercial PLA blend (80% PLA, 20% additives)	<2 mm	37	Mesophilic digestate from a full-scale dry anaerobic digester treating OFMSW	40	421.3						90.1	Hydrothermal (1 g VS-PLA, T = 200 °C, 60 min, 10 mL water)	CH_4_					
PLA	Commercial PLA blend (80% PLA, 20% additives)	<2 mm	37	Mesophilic digestate from a full-scale dry anaerobic digester treating OFMSW	40	442						94.5	Hydrothermal (1 g VS-PLA, T = 200 °C, 120 min, 10 mL 1% NaOH)	CH_4_					
PLA	Commercial PLA blend (80% PLA, 20% additives)	<2 mm	37	Mesophilic digestate from a full-scale dry anaerobic digester treating OFMSW	40	460.1						98.4	Hydrothermal (1 g VS-PLA, T = 240 °C, 10 min, 10 mL 10% NaOH)	CH_4_					
PLA	Commercial PLA blend (80% PLA, 20% additives)	<2 mm	37	Mesophilic digestate from a full-scale dry anaerobic digester treating OFMSW	40	449.8						96.2	Hydrothermal (1 g VS-PLA, T = 240 °C, 30 min, 10 mL 5% NaOH)	CH_4_					
PLA	Commercial PLA blend (80% PLA, 20% additives)	<2 mm	37	Mesophilic digestate from a full-scale dry anaerobic digester treating OFMSW	40	396.4						84.8	Hydrothermal (1 g VS-PLA, T = 240 °C, 60 min, 10 mL 1% NaOH)	CH_4_					
PLA	Commercial PLA blend (80% PLA, 20% additives)	<2 mm	37	Mesophilic digestate from a full-scale dry anaerobic digester treating OFMSW	40	351.5						75.2	Hydrothermal (1 g VS-PLA, T = 240 °C, 120 min, 10 mL water)	CH_4_					
PLA	PLA bags	10 × 10 mm film	37	Inoculum: anaerobic sludge from an anaerobic digester treating municipal wastewater	180	25.2						2.3 *		Biogas		SEM			[121]
PLA	PLA film	1–2 mm, thickness 80 μm		Inoculum: mesophilic digestate from a UASB anaerobic digester treating drink production effluents	60	5	34							Biogas		SEM			[147]
PLA	PLA film	1–2 mm, thickness 80 μm	Not spec.	Inoculum: mesophilic digestate from a UASB anaerobic digester treating drink production effluents	60	148.3	230						Alkaline (1 g PLA, 10 mL 0.5 M NaOH, 2.5 d, room T)	Biogas		SEM			
PLA	PLA film	3–5 mm, thickness 80 μm	30	Inoculum: mesophilic digestate from a UASB anaerobic digester treating drink production effluents	90		58.28					5.5		Biogas		SEM			
PLA	PLA film	3–5 mm, thickness 80 μm	30	Inoculum: mesophilic digestate from a UASB anaerobic digester treating drink production effluents	90		126.72					8.7 *	Thermal (45 °C, 12 h)	Biogas		SEM			
PLA	PLA film	3–5 mm, thickness 80 μm	30	Inoculum: mesophilic digestate from a UASB anaerobic digester treating drink production effluents	90		125.21					8.8 *	Thermal (60 °C, 12 h)	Biogas		SEM			
PLA	PLA film	3–5 mm, thickness 80 μm	30	Inoculum: mesophilic digestate from a UASB anaerobic digester treating drink production effluents	90		164.74					11.3 *	Thermal + alkaline (45 °C, 0.5 M NaOH, 10% *w*/*v* PLA, 12 h)	Biogas		SEM			
PLA	PLA film	3–5 mm, thickness 80 μm	30	Inoculum: mesophilic digestate from a UASB anaerobic digester treating drink production effluents	90		212.86					15.0 *	Thermal + alkaline (60 °C, 0.5 M NaOH, 10% *w*/*v* PLA, 12 h)	Biogas		SEM			
PLA	PLA film	3–5 mm, thickness 80 μm	30	Inoculum: mesophilic digestate from a UASB anaerobic digester treating drink production effluents	90		215.47					20.2 *	Thermal + alkaline (60 °C, 0.5 M NaOH, 10% *w*/*v* PLA, 24 h)	Biogas		SEM			
PLA	PLA film	3–5 mm, thickness 80 μm	30	Inoculum: mesophilic digestate from a UASB anaerobic digester treating drink production effluents	90		230.21					21.6 *	Thermal + alkaline (45 °C, 0.25 M NaOH, 10% *w*/*v* PLA, 32.2 h)	Biogas		SEM			
PLA	PLA film	3–5 mm, thickness 80 μm	30	Inoculum: mesophilic digestate from a UASB anaerobic digester treating drink production effluents	90		126.15					11.8 *	Thermal + alkaline (20 °C, 0.25 M NaOH, 10% *w*/*v* PLA, 12 h)	Biogas		SEM			
PLA	PLA film	3–5 mm, thickness 80 μm	30	Inoculum: mesophilic digestate from a UASB anaerobic digester treating drink production effluents	90		132.42					12.4 *	Thermal + alkaline (45 °C, 0.25 M NaOH, 10% w/v PLA, 12 h)	Biogas		SEM			
PLA	PLA film	3–5 mm, thickness 80 μm	30	Inoculum: mesophilic digestate from a UASB anaerobic digester treating drink production effluents	90		147.14					13.8 *	Thermal + alkaline (70 °C, 0.25 M NaOH, 10% *w*/*v* PLA, 12 h)	Biogas		SEM			
PLA	Commercial PLA items	2 mm	37	ISR=2 (VS basis)	250	130								CH_4_					[114]
PLA	Commercial PLA items	2 mm	37	ISR=2 (VS basis)	250	125							48 h, acidic pretreatment (HCl) to pH = 2	CH_4_					
PLA	Commercial PLA items	2 mm	37	ISR=2 (VS basis)	250	101							48 h, alkaline pretreatment (NaOH) to pH = 12	CH_4_					
PLA	Crystalline PLA	cups, 2 × 2 cm	37	Inoculum: anaerobic digestate from a digester treating wastewater	70			687						CH_4_	98.2				[148]
PLA	Crystalline PLA	cups, 2 × 2 cm	37	Inoculum: anaerobic digestate from a digester treating wastewater	70			928					Alkaline pretreatment (NaOH), 21 °C, pH = 12.96, 15 d	CH_4_					
PLA	NaturePlast	1 mm sheet	38	I/S = 2.85 (VS basis); working V = 300 mL	500	438						80.3		CH_4_				x	[95]
PLA	Total Corbion	1 mm sheet	38	I/S = 2.85 (VS basis); working V = 300 mL	500	344.4						74.7		CH_4_				x	
PLA	Commercial spoons	2–5 mm	38		49	63.4								CH_4_		FTIR, DSC			[149]
PLA	NaturePlast	Granules	38	BMP tests with I/S = 2.85 (VS basis)	520	429						82		CH_4_		SEM			[150]
PLA	NaturePlast	1–2 mm	38	BMP tests with I/S = 2.85 (VS basis)	520	427						82		CH_4_		SEM			
PLA	NaturePlast	0.8–1 mm	38	BMP tests with I/S = 2.85 (VS basis)	520	441						84		CH_4_		SEM			
PLA	NaturePlast	0.5–0.8 mm	38	BMP tests with I/S = 2.85 (VS basis)	520	441						84		CH_4_		SEM			
PLA	NaturePlast	0.3–0.5 mm	38	BMP tests with I/S = 2.85 (VS basis)	520	455						87		CH_4_		SEM			
PLA	NaturePlast	0.05–0.3 mm	38	BMP tests with I/S = 2.85 (VS basis)	520	460						88		CH_4_		SEM			
PLA	NaturePlast	Granules	38	BMP tests with I/S = 2.85 (VS basis)	25	14						3		CH_4_		SEM			
PLA	NaturePlast	Granules	38	BMP tests with I/S = 2.85 (VS basis)	25	389						75	150 °C 6 h	CH_4_		SEM			
PLA	NaturePlast	Granules	38	BMP tests with I/S = 2.85 (VS basis)	25	382						73	150 °C + 5% Ca(OH)_2_ 1 h	CH_4_		SEM			
PLA	NaturePlast	Granules	38	BMP tests with I/S = 2.85 (VS basis)	25	370						71	120 °C 24 h	CH_4_		SEM			
PLA	NaturePlast	Granules	38	BMP tests with I/S = 2.85 (VS basis)	25	391						75	120 °C + 5% Ca(OH)_2_ 6 h	CH_4_		SEM			
PLA	NaturePlast	Granules	38	BMP tests with I/S = 2.85 (VS basis)	25	147						28	90 °C 48 h	CH_4_		SEM			
PLA	NaturePlast	Granules	38	BMP tests with I/S = 2.85 (VS basis)	25	351						67	90 °C + 5% Ca(OH)_2_ 48 h	CH_4_		SEM			
PLA	NaturePlast	Granules	38	BMP tests with I/S = 2.85 (VS basis)	25	24						5	70 °C 48 h	CH_4_		SEM			
PLA	NaturePlast	Granules	38	BMP tests with I/S = 2.85 (VS basis)	25	328						63	70 °C + 5% Ca(OH)_2_ 48 h	CH_4_		SEM			
PLA	NaturePlast	Granules	38	BMP tests with I/S = 2.85 (VS basis)	30	21						4		CH_4_		SEM			
PLA	NaturePlast	Granules	38	BMP tests with I/S = 2.85 (VS basis)	30	136						26	90 °C 48 h	CH_4_		SEM			
PLA	NaturePlast	Granules	38	BMP tests with I/S = 2.85 (VS basis)	30	354						68	90 °C + 5% Ca(OH)_2_ 48 h	CH_4_		SEM			
PLA	NaturePlast	Granules	38	BMP tests with I/S = 2.85 (VS basis)	30	352						67	90 °C + 2.5% Ca(OH)_2_ 48 h	CH_4_		SEM			
PLA	NaturePlast	Granules	38	BMP tests with I/S = 2.85 (VS basis)	30	260						50	90 °C + 1.25% Ca(OH)_2_ 48 h	CH_4_		SEM			
PLA	NaturePlast	Granules	38	BMP tests with I/S = 2.85 (VS basis)	30	178						34	90 °C + 0.5% Ca(OH)_2_ 48 h	CH_4_		SEM			
PLA	NaturePlast	Granules	38	BMP tests with I/S = 2.85 (VS basis)	30	48						9	70 °C 48 h	CH_4_		SEM			
PLA	NaturePlast	Granules	38	BMP tests with I/S = 2.85 (VS basis)	30	338						65	70 °C + 5% Ca(OH)_2_ 48 h	CH_4_		SEM			
PLA	NaturePlast	Granules	38	BMP tests with I/S = 2.85 (VS basis)	30	381						73	70 °C + 2.5% Ca(OH)_2_ 48 h	CH_4_		SEM			
PLA	NaturePlast	Granules	38	BMP tests with I/S = 2.85 (VS basis)	30	286						55	70 °C + 1.25% Ca(OH)_2_ 48 h	CH_4_		SEM			
PLA	NaturePlast	Granules	38	BMP tests with I/S = 2.85 (VS basis)	30	167						32	70 °C + 0.5% Ca(OH)_2_ 48 h	CH_4_		SEM			
PLA	PLA (NaturePlast)	particles 1.01 mm (mean size)	38	I/S = 10 (VS basis)	400	426						82		CH_4_					[99]
PLA	PLA (NaturePlast)	particles 1.01 mm (mean size)	38	I/S = 4 (VS basis)	400	385						74		CH_4_					
PLA	PLA (NaturePlast)	particles 1.01 mm (mean size)	38	I/S = 2.85 (VS basis)	400	401						77		CH_4_					
PLA	PLA (NaturePlast)	particles 1.01 mm (mean size)	38	I/S = 2 (VS basis)	400	417						80		CH_4_					
PLA	PLA (NaturePlast)	particles 1.01 mm (mean size)	38	I/S = 1 (VS basis)	400	404						77		CH_4_					
PLA		0.1–0.25 mm	36	Anaerobic aqueous conditions ISO 14853; working V = 1 L; 1 gTS/L inoculum + 150 mg/L test material	77							4.6		Biogas					[123]
PLA		1.1 × 4.5 × 1.2 mm	35	Working V = 150 mL; polymer = 4.151 mg C/L	140	0						0		Biogas	0	FTIR, DSC, SEM			[137]
PLA	PLA (crystallinity 35%)		35		170	0		0				0		CH_4_					[151]
PLA	PLA (crystallinity 50%)		35		170	0		0				0		CH_4_					
PLA	PLA (amorphous)		35		170			189				40		CH_4_					
PLA blend	Ecovio^®^ (PLA + fossil biodegradable Ecoflex^®^ plastic) coffee capsules	<1 mm	38	Inoculum: sludge from a wastewater treatment plant, acclimated in the lab at 38 °C. Digestion conditions: ISR = 2.7 (VS basis), VS content = 9 g/L	100	127						24		X					[95]
PLA/PCL	PLA/PCL (80/20)	0.1–0.25 mm	36	Method ISO 14853; working V = 1 L; 1 g TS/L inoculum + 150 mg/L test material	77							0		Biogas					[123]
PLA+PBS	PLA/PBS (80/20)	<2 × 2 cm	35	Inoculum: sludge from a WWTP. Method: ISO 14853	56							0		Biogas		DSC, SEM			[126]
PLA+PCL	PLA/PCL (80/20)	<2 × 2 cm	35	Inoculum: sludge from a WWTP. Method: ISO 14853	56							0		Biogas		DSC, SEM			
PLA+PHB	PLA/PHB (80/20)	<2 × 2 cm	35	Inoculum: sludge from a WWTP. Method: ISO 14853	56							0		Biogas		DSC, SEM			
PLA+PHO	PLA/PHO (80/15)	<2 × 2 cm	35	Inoculum: sludge from a WWTP. Method: ISO 14853	56							2		Biogas		DSC, SEM			
PVA	Film	0.25 × 0.25 cm	35	ASTM D 5210-91; 150 mL working V + 100 mg polymer; flushed with N2	77							8		Biogas					[131]
PVA	Film	0.25 × 0.25 cm	35	ISO 11734; 150 mL working V+ 100 mg polymer; flushed with N_2_	77							10		Biogas					
PVA	PVA (Dupont)	5 × 5 × 1 mm film	38	Plastic: 2 g. Inoculum: supernatant from a laboratory scale digester fed with a mixture of primary domestic sludge and food waste	100						5			---	---		---		[152]
Starch-based	Vegemat^®^ coffee capsules	<1 mm	38	Inoculum: sludge from a wastewater treatment plant, acclimated in the lab at 38 °C. Digestion conditions: ISR = 2.7 (VS basis), VS content = 9 g/L	100	92						18		CH_4_				x	[95]
Starch blend	Starch (25% amylose) and PVA blend	Film	35	Plastic: 20 g. Inoculum: digestate from a wastewater treatment plant. Method: ASTM D5210-92.	25							52							[153]
Starch blend	High-amylose starch (80% amylose)-PVA blend	Film	35	Plastic: 20 g. Inoculum: digestate from a wastewater treatment plant. Method: ASTM D5210-92.	20							54							
Starch blend	Starch (from wheat)/PVOH	Foam	37	Substrate to inoculum ratio: 1 (VS basis). Inoculum: digestate from a mesophilic anaerobic digester	10	270						72.1		CH_4_					[154]
Starch blend	Starch (from potato)/PVOH	Foam	37	Substrate to inoculum ratio: 1 (VS basis). Inoculum: digestate from a mesophilic anaerobic digester	10	265						68.6		CH_4_					
Starch blend	Starch (from maize)/PVOH	Foam	37	Substrate to inoculum ratio: 1 (VS basis). Inoculum: digestate from a mesophilic anaerobic digester	10	248						75.4		CH_4_					
Starch blend	Starch:PVOH blends (90/10%)	5 × 5 × 1 mm film	38	Plastic: 2 g. Inoculum: supernatant from a laboratory scale digester fed with a mixture of primary domestic sludge and food waste	100						140								[152]
Starch blend	Starch:PVOH blends (75/25%)	5 × 5 × 1 mm film	38	Plastic: 2 g. Inoculum: supernatant from a laboratory scale digester fed with a mixture of primary domestic sludge and food waste	100						118								
Starch blend	Starch:PVOH blends (50/50%)	5 × 5 × 1 mm film	38	Plastic: 2 g. Inoculum: supernatant from a laboratory scale digester fed with a mixture of primary domestic sludge and food waste	100						60								
Starch blend	Starch-based film blend 1	1 × 1 cm film	37	Plastic to inoculum ratio: 0.25 (VS basis). Inoculum: digestate from a mesophilic digester treating municipal wastewater sludge	65							18.3			18.0				[115]
Starch blend	Starch-based film blend 2	1 × 1 cm film	37	Plastic to inoculum ratio: 0.25 (VS basis). Inoculum: digestate from a mesophilic digester treating municipal wastewater sludge	65							10.2			10.6				
Starch blend	Starch-based blend	4.3 mm	37	ISR: 4 (VS basis). Inoculum: digestate from a mesophilic lab-scale digester	26	250						35.9 ^●^		CH_4_					[155]
Starch blend	Starch-based blend	0.72 mm	37	ISR: 4 (VS basis). Inoculum: digestate from a mesophilic lab-scale digester	26	246						35.4 ^●^		CH_4_					
Starch blend	Starch-based blend	4.3 mm	37	ISR: 3 (VS basis). Inoculum: digestate from a mesophilic lab-scale digester	26	197						28.3 ^●^		CH_4_					
Starch blend	Starch-based blend	0.72 mm	37	ISR: 3 (VS basis). Inoculum: digestate from a mesophilic lab-scale digester	26	186						26.7 ^●^		CH_4_					
Starch blend	Starch-based blend	7.87 mm	37	ISR: 4 (VS basis). Inoculum: digestate from a mesophilic lab-scale digester	26	182						26.2 ^●^		CH_4_					
Starch blend	Starch-based blend	7.87 mm	37	ISR: 3 (VS basis). Inoculum: digestate from a mesophilic lab-scale digester	26	161						23.1 ^●^		CH_4_					
Starch blend	Starch-based blend	4.3 mm	37	ISR: 2 (VS basis). Inoculum: digestate from a mesophilic lab-scale digester	26	166						23.9 ^●^		CH_4_					
Starch blend	Starch-based blend	0.72 mm	37	ISR: 2 (VS basis). Inoculum: digestate from a mesophilic lab-scale digester	26	157						22.6 ^●^		CH_4_					
Starch blend	Starch-based blend	7.87 mm	37	ISR: 2 (VS basis). Inoculum: digestate from a mesophilic lab-scale digester	26	135						19.4 ^●^		CH_4_					
Starch blend	Starch-based shopping bags	film, 5 × 5 cm	37	Mesophilic digestate from a full-scale dry anaerobic digester treating OFMSW	60	119						29.5		CH_4_		FTIR			[10]
Starch blend	Starch-based shopping bags	film, 5 × 5 cm	37	Mesophilic digestate from a full-scale dry anaerobic digester treating OFMSW	90									CH_4_	67.3	FTIR			
Starch blend	Commercial spoons	2–5 mm	38		49	50.38								CH_4_		FTIR, DSC			[149]
starch blend	Granulate	0.2–0.63 mm	35	ASTM D 5210-91; 150 mL working V + 100 mg polymer; flushed with N_2_	41							57		Biogas					[131]
starch blend	Granulate	0.2–0.63 mm	35	ASTM D 5210-91; 150 mL working V + 100 mg polymer; flushed with 70% N_2_/30% CO_2_	33							55		Biogas					
starch blend	Granulate	0.2–0.63 mm	35	ISO 11734; 150 mL working V+ 100 mg polymer; flushed with N_2_	41							54.6		Biogas					
starch blend	Granulate	0.2–0.63 mm	35	ISO 11734; 150 mL working V+ 100 mg polymer; flushed with 70% N_2_/30% CO_2_	33							49		Biogas					
Starch-based	Starch-based bags	2 mm	37	ISR = 2 (VS basis)	250	200.9								CH_4_					[114]
Starch-based	Starch-based bags	2 mm	37	ISR = 2 (VS basis)	250	203.9							48 h, acidic pretreatment (HCl) to pH = 2	CH_4_					
Starch-based	Starch-based bags	2 mm	37	ISR = 2 (VS basis)	250	158							48 h, alkaline pretreatment (NaOH) to pH = 12	CH_4_					
Starch-based	Starch-based cutlery	2 mm	37	ISR = 2 (VS basis)	250	312.5								CH_4_					
Starch-based	Starch-based cutlery	2 mm	37	ISR = 2 (VS basis)	250	302.5							48 h, acidic pretreatment (HCl) to pH = 2	CH_4_					
Starch-based	Starch-based cutlery	2 mm	37	ISR=2 (VS basis)	250	252.9							48 h, alkaline pretreatment (NaOH) to pH = 12	CH_4_					
TPS	TPS (Bioplast TPS, BIOTEC)	<2 × 2 cm	35	Inoculum: sludge from a WWTP. Method: ISO 14853	56							98%		biogas		DSC, SEM			[126]
TPS	TPS	1 mm sheet	38	I/S = 2.85 (VS basis); working V = 300 mL	30	309.5						82.6%		CH_4_				x	[95]

* Biodegradability evaluated from total biogas production. ^●^ Biodegradability data recalculated from the data provided in the manuscript. ^(1)^ L CH_4_/kg VS; ^(2)^ L biogas/kg VS; ^(3)^ L CH_4_/kg polymer; ^(4)^ L biogas/kg polymer; ^(5)^ L CH_4_/kg ThOD; ^(6)^ L biogas/kg COD.

**Table A2:** Summary of literature results related to anaerobic degradation of different bioplastic products under thermophilic conditions (expanded from [93,109]).

Class	Bioplastic Type	Size and Shape	T	Test Conditions	Time	Biogas/Methane Production				Degree of Biodegr.	Pre-Treatment	Biodegr. Eval.	Mass Loss	Analytical Techniques	Visual Insp.	Microb. Charact.	Ref.
			(°C)			^(1)^	^(2)^	^(3)^	^(4)^	(%)			(%)				
Cellulose-based	Cellulose	1 × 1 cm film	55	Plastic to inoculum ratio: 0.5. Inoculum: sludge from a waste management company	35		280			18.3		biogas			x		[156]
Cellulose-based	Cellulose	2 × 2 cm film	55	Plastic to inoculum ratio: 0.5. Inoculum: sludge from a waste management company	35		260			17.1		biogas			x		
Cellulose-based	Cellulose	3 × 3 cm film	55	Plastic to inoculum ratio: 0.5. Inoculum: sludge from a waste management company	35		250			16.3		biogas			x	x	
Starch-based	Vegemat^®^ coffee capsules	<1 mm	58	Inoculum: sludge from a wastewater treatment plant, acclimated in the lab at 58 °C. Digestion conditions: ISR = 2.7 (VS basis), VS content = 9 g/L	100	355				69		CH_4_					[95]
Mater-Bi	Mater-Bi coffee capsules	<1 mm	58	Inoculum: sludge from a wastewater treatment plant, acclimated in the lab at 58 °C. Digestion conditions: ISR = 2.7 (VS basis), VS content = 9 g/L	100	257				47		CH_4_				x	
Mater-Bi	Mater-Bi (60% starch, 40% hydrophilic resin)	entire bag	55	Plastic to inoculum ratio: 0.5 (VS basis). Inoculum: liquid digestate from mesophilic anaerobic digester fed with manure, agro-wastes, and residues shifted progressively to thermophilic condition	30	186						CH_4_	28.5		x		[116]
Mater-Bi	Mater-Bi (PCL + starch, Novamont)	Small piece of plastic bags <1 mm	55	Plastic: 1 g. Inoculum: 5 mL of pig slurry mixed with synthetic medium for methanogens and acclimated to mesophilic anaerobic condition	90	303				55		---			x		[12]
Mater-Bi	Shopper	2.5 × 2.5 cm	55	300 mL inoculum + 3 g bioplastic	15	95				22.5		CH_4_	21.7	FTIR	x		[157]
Mater-Bi	Shopper	2.5 × 2.5 cm	55	300 mL inoculum + 3 g bioplastic	30	139				25.5		CH_4_	28.7	FTIR	x		
Mater-Bi	Shopper	2.5 × 2.5 cm	55	300 mL inoculum + 3 g bioplastic	60	165				29.2		CH_4_	30.0	FTIR	x		
Mater-Bi	Shopper	2.5 × 2.5 cm	55	300 mL inoculum + 3 g bioplastic	30	142				25.1		CH_4_	26.8	FTIR	x		[158]
Mater-Bi	Shopper	2.5 × 2.5 cm	55	300 mL inoculum + 3 g bioplastic	60	194				34.4		CH_4_	35.0	FTIR	x		
Mater-Bi	Shopper	2.5 × 2.5 cm	55	300 mL inoculum + 3 g bioplastic	90	224				40		CH_4_	37.8	FTIR	x		
PBAT	Commercial PBAT	2 × 2 mm, thickness 0.1 mm	52	Inoculum: mixture of soil (70%) and anaerobic sludge (30%) from a municipal wastewater treatment plant. PBAT addition: 1% wt.	75							---	9.3	SEM	x		[159]
PBAT	PBAT 93 000 g/mol (Ecoflex, BASF)	5 × 5 mm film 70 μm of thickness	55	Inoculum: mesophilic anaerobic sludge (37 °C) from a municipal waste water-treatment plant acclimated to thermophilic temperature (55 °C) for two weeks	126					8.3		biogas	8.5	DSC, XRD			[122]
PBAT	PBAT	1 mm sheet	58	I/S = 2.85 (VS basis); working volume = 300 mL	100	11.05				1.7		CH_4_				x	[95]
PBS	Commercial PBS	2 × 2 mm, thickness 0.1 mm	52	Inoculum: mixture of soil (70%) and anaerobic sludge (30%) from a municipal wastewater treatment plant. PBS addition: 1% wt.	75							---	36.2	SEM	x		[159]
PBS	PBS (PBE 003, NaturePlast	<2 × 2 cm	55	Method: high solid anaerobic digestion (ISO 15985)	90					12		biogas		DSC, SEM			[126]
PBS	PBS (Enpol G4560, IRE Chemical Ltd.)	5 × 5 mm thin film (100 μm)	55	Plastic: 50 mg. Inoculum: mesophilic anaerobic sludge from a wastewater treatment plant acclimated to thermophilic temperature	113					20.2		biogas		DSC, XRD, SEM			[127]
PBS	PBS (Enpol G4560, IRE Chemical Ltd.)	5 × 5 mm thick film (1.02 mm)	55	Plastic: 50 mg. Inoculum: mesophilic anaerobic sludge from a wastewater treatment plant acclimated to thermophilic temperature	113					20.1		biogas	24.8	DSC, XRD, SEM			
PBS	PBS (Enpol G4560, IRE Chemical Ltd.)	Powder (320 μm)	55	Plastic: 50 mg. Inoculum: mesophilic anaerobic sludge from a wastewater treatment plant acclimated to thermophilic temperature	113					18.1		biogas		DSC, XRD, SEM			
PBS	PBS (Enpol G4560, IRE Chemical Ltd.)	5 × 5 mm thin film (100 μm)	55	Plastic: 50 mg. Inoculum: mesophilic anaerobic sludge from a wastewater treatment plant shifted to thermophilic temperature with addition of a PBS acclimated inoculum from a previous experiment	113					23.3		biogas		DSC, XRD, SEM			
PBS	PBS (Enpol G4560, IRE Chemical Ltd.)	5 × 5 mm thick film (1.02 mm)	55	Plastic: 50 mg. Inoculum: mesophilic anaerobic sludge from a wastewater treatment plant shifted to thermophilic temperature with addition of a PBS acclimated inoculum from a previous experiment	113					22		biogas	25.4	DSC, XRD, SEM			
PBS	PBS (Enpol G4560, IRE Chemical Ltd.)	Powder (320 μm)	55	Plastic: 50 mg. Inoculum: mesophilic anaerobic sludge from a wastewater treatment plant shifted to thermophilic temperature with addition of a PBS acclimated inoculum from a previous experiment	113					10.3		biogas		DSC, XRD, SEM			
PBS	PBS (Sigma-Aldrich)	125–250 μm	55	Plastic: 10 g. Inoculum: digestate from a mesophilic anaerobic digester treating cow manure and green waste acclimated to 55 °C. Pre-incubation of the inoculum with 20 mL of sludge acclimated to PLA	100					3		biogas				x	[101]
PBS	PBS	1 mm sheet	58	I/S = 2.85 (VS basis); working volume = 300 mL	100	0				0		CH_4_				x	[95]
PCL	PCL (Mn 58.1 kg.mol^−1^)	10 × 10 × 0.7 mm film	55	Plastic to inoculum ratio: 0.38 g COD/g VSS. Inoculum: thermophilic digested sludge from a digester	140				663	60		biogas		DSC, SEM			[160]
PCL	PCL (Mn 38. kg.mol^−1^)	Powder	55		80				643	54		biogas		DSC, SEM			
PCL	PCL (Mn 13 kg.mol^−1^)		55		70				676	57		biogas		DSC, SEM			
PCL	PCL (CAPA 6500, Perstorp)	<2 × 2 cm	55	Method: high solid anaerobic digestion (ISO 15985)	127					95		biogas		DSC, SEM			[126]
PCL	PCL (Mw 65,000, Aldrich)	125–250 μm	55	Plastic: 10 g. Inoculum: digestate from a mesophilic anaerobic digester treating cow manure and green waste acclimated to 55 °C	47			697		92 *		biogas					[161]
PCL	PCL (Sigma-Aldrich)	125–250 μm	55	Plastic: 10 g. Inoculum: digestate from a mesophilic anaerobic digester treating cow manure and green waste acclimated to 55 °C. Pre-incubation of the inoculum with 20 mL of sludge acclimated to PLA	45					84		biogas				x	[101]
PCL	PCL	1-cm^2^ film	52	Plastic to inoculum ratio: 0.5 (VS basis). Inoculum: digestate from a mesophilic anaerobic digester fed with food wastes and manure shifted to thermophilic temperature (10 days)	30	44.4				11.3		CH_4_					[130]
PCL	PCL	1 mm sheet	58	I/S = 2.85 (VS basis); working volume = 300 mL	100	0				0		CH_4_				x	[95]
PCL	PCL (Mw 65,000, Aldrich)	<125 μm	55	Plastic: 10 g. Inoculum: digestate from a mesophilic anaerobic digester treating cow manure and green waste acclimated to 55 °C	38.5					88 *	Size red.	biogas					[161]
PCL	PCL (Mw 65,000, Aldrich)	125–250 μm	55	Plastic: 10 g. Inoculum: digestate from a mesophilic anaerobic digester treating cow manure and green waste acclimated to 55 °C	58.5					85 *	Size red.	biogas					
PCL	PCL (Mw 65,000, Aldrich)	250–500 μm	55	Plastic: 10 g. Inoculum: digestate from a mesophilic anaerobic digester treating cow manure and green waste acclimated to 55 °C	65					81 *	Size red.	biogas					
PCL+PHO	PCL/PHO (85/15)	<2 × 2 cm	55	Method: high solid anaerobic digestion (ISO 15985)	66					85		biogas		DSC, SEM			[126]
PCL+TPS	PCL/TPS (70/30)	<2 × 2 cm	55	Method: high solid anaerobic digestion (ISO 15985)	80					68		biogas		DSC, SEM			
PCL+TPS	80% PCL 20% TPS	1-cm^2^ film	52	Plastic to inoculum ratio: 0.5 (VS basis). Inoculum: digestate from a mesophilic anaerobic digester fed with food wastes and manure shifted to thermophilic temperature (10 days)	30	104				26.2		biogas		DSC, SEM			[130]
PHB	PHB (ENMAT Y1000, TiTAN)	<2 × 2 cm	55	Method: high solid anaerobic digestion (ISO 15985)	127					92		biogas		DSC, SEM			[126]
PHB	PHB (Sigma-Aldrich)	125–250 μm	55	Plastic: 10 g. Inoculum: digestate from a mesophilic anaerobic digester treating cow manure and green waste acclimated to 55 °C. Pre-incubation of the inoculum with 20 mL of sludge acclimated to PLA	18					88		biogas				x	[101]
PHB	PHB Biomer	1 mm sheet	58	I/S = 2.85 (VS basis); working V = 300 mL	45	350.8				57.6		CH_4_				x	[95]
PHB	PHB K. D.	1 mm sheet	58	I/S = 2.85 (VS basis); working V = 300 mL	49	399.1				72.3		CH_4_				x	
PHB+PBS	PHB/PBS (50/50)	<2 × 2 cm	55	Method: high solid anaerobic digestion (ISO 15985)	121					78		biogas		DSC, SEM			[126]
PHB+PCL	PHB/PCL (60/40)	<2 × 2 cm	55	Method: high solid anaerobic digestion (ISO 15985)	80					104		biogas		DSC, SEM			
PHB+PHO	PHB/PHO (85/15)	<2 × 2 cm	55	Method: high solid anaerobic digestion (ISO 15985)	66					87		biogas		DSC, SEM			
PHBV	PHBV	Pellets	55	Inoculum: 1:1 mixture of mesophilic and thermophilic digestate from lab-scale digesters. ISR = 1 (VS basis). Solids content in the reactor: 7.22% TS	104	80.5						---		SEM			[142]
PHBV	Commercial PHBV	2 × 2 mm, thickness 0.1 mm	52	Inoculum: mixture of soil (70%) and anaerobic sludge (30%) from a municipal wastewater treatment plant. PHBV addition: 1% wt.	75							---	100.0	SEM	x		[159]
PHO	PHO (Bioplastech R, Bioplastech)	<2 × 2 cm	55	Method: high solid anaerobic digestion (ISO 15985)	50					6		biogas		DSC, SEM			[126]
PLA	Commercial PLA blend (80% PLA, 20% additives)	<2 mm	55	Mesophilic digestate from a full-scale anaerobic digestere treating sewage sludge	146	442.6				94.8		CH_4_					[146]
PLA	Commercial PLA	2 × 2 mm, thickness 0.1 mm	52	Inoculum: mixture of soil (70%) and anaerobic sludge (30%) from a municipal wastewater treatment plant. PLA addition: 1% wt.	75							---	60.0	SEM	x		[159]
PLA	PLA (Mn 44.5 kg/mol)	10 × 10 × 0.7 mm film	55	Plastic to inoculum ratio: 0.15 g COD/g VSS. Inoculum: thermophilic digested sludge from a digester	120				677	74		biogas		DSC, SEM			[160]
PLA	PLA (Mn 3.4 kg/mol)	Powder	55	Plastic to inoculum ratio: 0.15 g COD/g VSS. Inoculum: thermophilic digested sludge from a digester	90				520	56		biogas		DSC, SEM			
PLA	PLA (Mn 0.35 kg/mol)	Powder	55	Plastic to inoculum ratio: 0.15 g COD/g VSS. Inoculum: thermophilic digested sludge from a digester	30				625	84		biogas		DSC, SEM			
PLA	PHB (Biopol)	2 × 2 cm	52	Plastic: 3–5 g. Inoculum: anaerobic digester for solid waste	20					73		biogas					[144]
PLA	PLA	2 × 2 cm	52	Plastic: 3–5 g. Inoculum: anaerobic digester for solid waste	40					60		biogas					
PLA	PLA	1 × 1, 2 × 2, 3 × 3 cm rigid pieces	55	Plastic to inoculum ratio: 0.5. Inoculum: sludge from a waste management plant	35		20			0		biogas			x		[156]
PLA	PLA (Luminy L130, Mw = 130 kDa)	Pellets	55	Plastic: 3 g. Inoculum: sludge from a thermophilic anaerobic digester treating food waste, plant residues, and other organic waste products	104			224				CH_4_	70.0			x	[102]
PLA	PLA (Luminy L175, Mw = 175 kDa)	Pellets	55	Plastic: 3 g. Inoculum: sludge from a thermophilic anaerobic digester treating food waste, plant residues, and other organic waste products	104			266				CH_4_	77.7				
PLA	PLA (Biopolymer-4043D, Nature Works)	<2 × 2 cm	55	Plastic: 15 g. Inoculum: 1 kg of digestate from a thermophilic reactor treating household waste.	80					88		biogas		DSC, SEM			[126]
PLA	PLA film 25 μm of thickness (Unitaka)	Powder 125–250 μm	55	Inoculum: digestate from a mesophilic anaerobic digester treating cow manure and green waste acclimated to 55 °C. Addition of 20 mL of acclimated sludge to PLA thermophilic digestion during the pre-incubation	73				782	84.1 *		biogas					[162]
PLA	PLA (H-400, Mitsui Chemical)	125–250 μm	55	Plastic: 10 g. Inoculum: digestate from a mesophilic anaerobic digester treating cow manure and green waste acclimated to 55 °C. Undiluted inoculum used	82			469		91 *		biogas					[161]
PLA	PLA (H-400, Mitsui Chemical)	125–250 μm	55	Plastic: 10 g. Inoculum: digestate from a mesophilic anaerobic digester treating cow manure and green waste acclimated to 55 °C. Diluted inoculum used	107			388		79 *		biogas					
PLA	PLA (H-400, Mitsui Chemical)	125–250 μm	55	Plastic: 5 g. Inoculum: digestate from a mesophilic anaerobic digester treating cow manure and green waste acclimated to 55 °C. Diluted inoculum used	112			374		80 *		biogas					
PLA	PLA (Ingeo)	Small piece of plastic bags <1 mm	55	Plastic: 1 g. Inoculum: 5 mL of pig slurry mixed with synthetic medium for methanogens and acclimated to mesophilic anaerobic condition	90	267				56		---			x		[12]
PLA	PLA (Fabri-Kal Inc.)	Plastic cup ground to 3 mm	58	Plastic: 1 g. Inoculum: 10 mL of anaerobic inoculum	56	187				40							[143]
PLA	PLA (Unitika)	125–250 μm	55	Plastic: 10 g. Inoculum: digestate from a mesophilic anaerobic digester treating cow manure and green waste acclimated to 55 °C. Pre-incubation of the inoculum with 20 mL of sludge acclimated to PLA	80					82		biogas				x	[101]
PLA	PLA (NatureWorks 4043D)	Sheets	52	Plastic to inoculum ratio: 0.5 (VS basis). Inoculum: digestate from a mesophilic anaerobic digester treating industrial food waste and manure	36	409				90		CH_4_					[163]
PLA	PLA (plastic cup)	2 × 2 × 0.5 mm	58	Plastic to inoculum ratio: 2–4 kg VS/m^3^. Inoculum: digestate from a mesophilic anaerobic digester treating wastewater treatment acclimated to 58 °C for 14 days. Method: EN ISO 11734:2003	60		835			90		biogas		FTIR, opt. microscopy			[145]
PLA	PLA (NaturePlast)	particles 1.01 mm (mean size)	58	I/S = 10 (VS basis)	100	456				87.3		CH_4_				x	[99]
PLA	PLA (NaturePlast)	particles 1.01 mm (mean size)	58	I/S = 4 (VS basis)	100	423				81.0		CH_4_				x	
PLA	PLA (NaturePlast)	particles 1.01 mm (mean size)	58	I/S = 2.85 (VS basis)	100	390				74.7		CH_4_				x	
PLA	PLA (NaturePlast)	particles 1.01 mm (mean size)	58	I/S = 2 (VS basis)	100	404				77.4		CH_4_				x	
PLA	PLA (NaturePlast)	particles 1.01 mm (mean size)	58	I/S = 1 (VS basis)	100	374				71.6		CH_4_				x	
PLA	cup	10 × 10 mm	55	untreated	100	453				97				FTIR, DSC, opt. microscopy			[82]
PLA	PLA (cutlery)	2.5 × 2.5 cm	55	300 mL inoculum + 3 g bioplastic	15	56				6.6		CH_4_	6.0	FTIR	x		[157]
PLA	PLA (dish)	2.5 × 2.5 cm	55	300 mL inoculum + 3 g bioplastic	15	44				6.1		CH_4_	7.8	FTIR	x		
PLA	PLA (cutlery)	2.5 × 2.5 cm	55	300 mL inoculum + 3 g bioplastic	30	154				21.5		CH_4_	23.3	FTIR	x		
PLA	PLA (dish)	2.5 × 2.5 cm	55	300 mL inoculum + 3 g bioplastic	30	108				19.1		CH_4_	19.7	FTIR	x		
PLA	PLA (cutlery)	2.5 × 2.5 cm	55	300 mL inoculum + 3 g bioplastic	60	168				29.8		CH_4_	29.2	FTIR	x		
PLA	PLA (dish)	2.5 × 2.5 cm	55	300 mL inoculum + 3 g bioplastic	60	123				24.9		CH_4_	24.2	FTIR	x		
PLA	PLA (cutlery)	2.5 × 2.5 cm	55	300 mL inoculum + 3 g bioplastic	30	104				18.4		CH_4_	16.4	FTIR	x		
PLA	PLA (dish)	2.5 × 2.5 cm	55	300 mL inoculum + 3 g bioplastic	30	81				14.3		CH_4_	17.9	FTIR	x		
PLA	PLA (cutlery)	2.5 × 2.5 cm	55	300 mL inoculum + 3 g bioplastic	60	279				49.4		CH_4_	52.0	FTIR	x		
PLA	PLA (dish)	2.5 × 2.5 cm	55	300 mL inoculum + 3 g bioplastic	60	215				38.1		CH_4_	43.9	FTIR	x		
PLA	PLA (cutlery)	2.5 × 2.5 cm	55	300 mL inoculum + 3 g bioplastic	90	397				70.3		CH_4_	72.1	FTIR	x		
PLA	PLA (dish)	2.5 × 2.5 cm	55	300 mL inoculum + 3 g bioplastic	90	330				58.4		CH_4_	61.1	FTIR	x	x	
PLA	NaturePlast	1 mm sheet	58	I/S = 2.85 (VS basis); working volume = 300 mL	58	389				74.6		CH_4_				x	[95]
PLA	Total Corbion	1 mm sheet	58	I/S = 2.85 (VS basis); working volume = 300 mL	98	335				74.6		CH_4_					
PLA	PLA film 25 μm of thickness (Unitaka)	Crushed film (>500 μm)	55	Inoculum: digestate from a mesophilic anaerobic digester treating cow manure and green waste acclimated to 55 °C. Addition of 20 mL of acclimated sludge to PLA thermophilic digestion during the pre-incubation	60				936	97.5	Size red.	biogas					[162]
PLA	PLA film 25 μm of thickness (Unitaka)	1 × 1 cm film, 25 μm of thickness	55	Inoculum: digestate from a mesophilic anaerobic digester treating cow manure and green waste acclimated to 55 °C. Addition of 20 mL of acclimated sludge to PLA thermophilic digestion during the pre-incubation	60				880	94.5	Size red.	biogas					
PLA	PLA film 25 μm of thickness (Unitaka)	15 × 34 cm film, 25 μm of thickness	55	Inoculum: digestate from a mesophilic anaerobic digester treating cow manure and green waste acclimated to 55 °C. Addition of 20 mL of acclimated sludge to PLA thermophilic digestion during the pre-incubation	60				893	96	Size red.	biogas					
PLA	PLA film 25 μm of thickness (Unitaka)	39 × 82 cm film, 25 μm of thickness	55	Inoculum: digestate from a mesophilic anaerobic digester treating cow manure and green waste acclimated to 55 °C. Addition of 20 mL of acclimated sludge to PLA thermophilic digestion during the pre-incubation	60				827	89	Size red.	biogas					
PLA	cup	10 × 10 mm	55		100	448				96	Hydrothermal pretreatment (2 h 90 °C)			FTIR, DSC, opt. microscopy			[82]
PLA	cup	10 × 10 mm	55		100	448				96	Alkaline pretreatment (2 h 0.1 M KOH, Tamb)			FTIR, DSC, opt. microscopy			
PLA	PLA	Commercial items	55	Plastic: 1 g. Inoculum: 10 mL of anaerobic inoculum	56	225				48.2	Steam exposition, 3 h 120 °C						[143]
PLA blend	80% PLA, 20% PBS (blend produced by mixing and melting the components)	Sheets	52	Plastic to inoculum ratio: 0.5 (VS basis). Inoculum: digestate from a mesophilic anaerobic digester treating industrial food waste and manure	60	190				37		CH_4_					[163]
PLA blend	70% PLA, 30% PCL (blend produced by mixing and melting the components)	Sheets	52	Plastic to inoculum ratio: 0.5 (VS basis). Inoculum: digestate from a mesophilic anaerobic digester treating industrial food waste and manure	60	297				63		CH_4_					
PLA blend	76% PLA, 19% PBS, 5% CaCO_3_ (Omya TP39914) (blend produced by mixing and melting the components)	Sheets	52	Plastic to inoculum ratio: 0.5 (VS basis). Inoculum: digestate from a mesophilic anaerobic digester treating industrial food waste and manure	60	210				45		CH_4_					
PLA blend	76% PLA, 19% PBS, 5% CaCO_3_ (Omya TP39968) (blend produced by mixing and melting the components)	Sheets	52	Plastic to inoculum ratio: 0.5 (VS basis). Inoculum: digestate from a mesophilic anaerobic digester treating industrial food waste and manure	60	230				49		CH_4_					
PLA blend	Ecovio^®^ (PLA + fossil biodegradable Ecoflex^®^ plastic) coffee capsules	<1 mm	58	Inoculum: sludge from a wastewater treatment plant, acclimated in the lab at 58 °C. Digestion conditions: ISR = 2.7 (VS basis), VS content = 9 g/L	100	308				58		CH_4_					[95]
PLA blend	PLA/PBS (80/20)	<2 × 2 cm	55	High-solids anaerobic digestion (ISO 15985). Inoculum: Digestate from an anaerobic digester treating the organic fraction of household waste and stabilized in a post-fermentation phase	121					84		biogas		DSC, SEM			[126]
PLA blend	PLA/PCL (80/20)	<2 × 2 cm	55	High-solids anaerobic digestion (ISO 15985). Inoculum: Digestate from an anaerobic digester treating the organic fraction of household waste and stabilized in a post-fermentation phase	121					90		biogas		DSC, SEM			
PLA blend	PLA/PHB (80/20)	<2 × 2 cm	55	High-solids anaerobic digestion (ISO 15985). Inoculum: Digestate from an anaerobic digester treating the organic fraction of household waste and stabilized in a post-fermentation phase	80					104		biogas		DSC, SEM			
PLA blend	PLA/PHO (80/15)	<2 × 2 cm	55	High-solids anaerobic digestion (ISO 15985). Inoculum: Digestate from an anaerobic digester treating the organic fraction of household waste and stabilized in a post-fermentation phase	66					90		biogas		DSC, SEM			
TPS	TPS (Bioplast TPS, BIOTEC)	<2 × 2 cm	55	High-solids anaerobic digestion (ISO 15985). Inoculum: Digestate from an anaerobic digester treating the organic fraction of household waste and stabilized in a post-fermentation phase	127					81		biogas		DSC, SEM			
TPS	TPS (70% starch from MP Biomedicals LLC and 30% glycerol)	1-cm^2^ film	52	Plastic to inoculum ratio: 0.5(VS basis). Inoculum: digestate from a mesophilic anaerobic digester fed with food wastes and manure shifted to thermophilic temperature (10 days)	30	32				77.1		CH_4_					[130]
TPS	TPS	1 mm sheet	58	I/S = 2.85 (VS basis); working volume = 300 mL	22	304				80.2		CH_4_				x	[95]

* Biodegradability evaluated from total biogas production. ^(1)^ L CH_4_/kg VS; ^(2)^ L biogas/kg VS; ^(3)^ L CH_4_/kg polymer; ^(4)^ L biogas/kg polymer.
